# Chemical Mechanical Polishing of Plasma‐Modified Cu/Polymer Interfaces for Advanced Hybrid Bonding

**DOI:** 10.1002/advs.202512611

**Published:** 2025-10-29

**Authors:** Sukkyung Kang, Chansu Jeon, Juseong Park, Seulah Park, Kyung Min Kim, Sanha Kim

**Affiliations:** ^1^ Department of Mechanical Engineering Korea Advanced Institute of Science and Technology (KAIST) Daejeon 34141 Republic of Korea; ^2^ InnoCORE PRISM‐AI Center Korea Advanced Institute of Science and Technology (KAIST) Daejeon 34141 Republic of Korea; ^3^ Department of Material Science and Engineering Korea Advanced Institute of Science and Technology (KAIST) Daejeon 34141 Republic of Korea

**Keywords:** argon plasma treatment, benzocyclobutene, chemical mechanical polishing, hybrid bonding

## Abstract

Hybrid bonding, referring to direct bonding between metal and insulator layers, has become a key technology for next‐generation semiconductor packaging, enabling high‐density interconnects, fast signal transmission, and a compact form factor. Polymer dielectrics such as benzocyclobutene (BCB) provide excellent insulation and adhesion but pose challenges for chemical mechanical polishing (CMP) due to their viscoelasticity. Here, a CMP‐compatible strategy based on argon (Ar) plasma modification of BCB is proposed to enable effective planarization of Cu/BCB hybrid bonding interfaces. Ar plasma treatment increases surface hardness and brittleness of half‐cured BCB and increases hydrophilicity. The resulting wrinkle structures improve the flow of abrasive particles and slurry, enabling a plowing‐dominant removal mechanism for precision planarization. Modified BCB shows a removal rate of up to 17.3 nm s^−1^, over five times higher than untreated BCB, while Cu surface roughness is reduced from 51.4 to 3.0 nm. A time‐dependent dishing model is developed to elucidate dishing mechanism and to control dishing geometry precisely. Using this model, a BCB dishing depth of 230 nm is achieved, supporting void‐free and reliable hybrid bonding. Overall, this study presents a simple yet practical approach to CMP of soft polymer dielectrics and offers a promising solution to advanced heterogeneous integration technologies.

## Graphical abstract

Hybrid bonding demands planarization of Cu/polymer interfaces, but soft polymers like benzocyclobutene (BCB) are difficult to polish by conventional chemical mechanical polishing (CMP). It is demonstrated that Ar plasma modification transforms BCB into a CMP‐compatible surface, enabling effective planarization and void‐free bonding. This simple yet effective approach provides a practical foundation for next‐generation packaging platforms, such as polymer interposers and fine‐pitch interconnects.

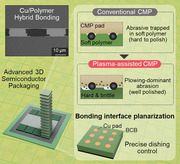

## 1 Introduction

Data‐centric artificial intelligence (AI) technologies have been demanding higher integration density and faster data transmission between the processing unit and memory.^[^ ^1^, ^2^, ^3^ ^]^ To meet these demands, advanced packaging technologies, such as high‐bandwidth memory (HBM) with 3D chip stacking, have become increasingly important.^[^ ^4^, ^5^, ^6^ ^]^ Hybrid bonding is a key technology for next‐generation 3D integration, enabling direct bonding of metal/dielectric interfaces without the use of micro‐bumps.^[^ ^7^, ^8^, ^9^, ^10^ ^]^ This approach significantly reduces interconnect pitch, increases the number of input/output (I/O) channels, and enhances data throughput.^[^ ^11^, ^12^ ^]^ Moreover, eliminating the bonding layer enables thinner packaging while improving power efficiency and reducing signal delay.^[^ ^13^, ^14^ ^]^

In conventional hybrid bonding, silicon dioxide (SiO_2_) has been widely adopted due to its high compatibility with conventional complementary metal‐oxide‐semiconductor (CMOS) fabrication.^[^ ^15^, ^16^, ^17^ ^]^ However, because hybrid bonding relies on thermal compression,^[^ ^18^, ^19^, ^20^ ^]^ brittle ceramic materials are prone to fracture or chipping under localized stress^[^ ^21^, ^22^, ^23^ ^]^ or by particles.^[^ ^24^, ^25^ ^]^ These failures often result in interfacial cracks or delamination, threatening long‐term reliability. In addition, ceramic‐to‐ceramic bonding typically involves both plasma and wet treatments to induce surface hydrophilicity,^[^ ^26^, ^27^, ^28^ ^]^ which can inadvertently lead to copper oxidation, severely compromising the electrical integrity of the interconnects.^[^ ^29^, ^30^ ^]^

As an alternative to these conventional technologies, Cu/polymer hybrid bonding technologies that utilize polymeric insulators, such as benzocyclobutene (BCB) and polyimide (PI), have been gaining increasing attention.^[^ ^31^, ^32^, ^33^ ^]^ Compared to ceramics, polymer‐based dielectrics exhibit greater mechanical flexibility and reduced brittleness, which helps alleviate physical stress during bonding.^[^ ^34^, ^35^ ^]^ In addition, the copper oxidation issue can be avoided since bonding is achieved by heat alone, unlike SiO_2_‐based processes that require oxidizing treatments for hydrophilic surfaces.^[^ ^36^, ^37^ ^]^ Also, their viscoelastic behavior near their glass transition temperature (Tg) enables the self‐adaptive deformation,^[^ ^38^ ^]^ which helps mitigate surface defects and increases tolerance to surface irregularities.^[^ ^39^ ^]^ In addition, polymers offer high compatibility with non‐CMOS technologies, making them suitable for more advanced packaging applications for wearable and flexible electronics.

**Figure 1:**
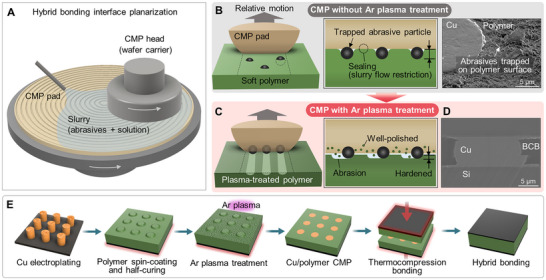
Ar plasma‐assisted chemical mechanical polishing (CMP) for Cu/polymer hybrid bonding. Illustrations showing A) the CMP setup used for planarization of hybrid bonding interfaces. B) Challenges in CMP for soft polymers, where abrasives are being trapped and slurry flow is restricted. The scanning electron microscope (SEM) image shows the abrasive particles trapped on the polymer surface after CMP. An illustration of C) effective abrasion and stable material removal during CMP enabled by the Ar plasma. D) Cross‐section of a successful Cu/BCB hybrid bonding interface using the planarized interfaces. E) Schematic overview of Ar plasma‐assisted CMP and hybrid bonding process.

Despite these advantages, precisely performing chemical mechanical polishing (CMP) on half‐cured polymers for hybrid bonding remains a significant challenge,^[^ ^40^ ^]^ representing one of the biggest hurdles to achieving reliable hybrid bonding. CMP removes material by abrasive particles dispersed in slurry, which are indented into the target surface under applied pressure and remove material through a plowing mechanism (**Figure** **1**A).^[^ ^41^, ^42^ ^]^ However, as both the half‐cured BCB and the porous polyurethane pads are highly compliant, abrasive particles are easily trapped between the two soft materials, forming a sealed interface and limiting the CMP process (Figure 1B).^[^ ^43^, ^44^ ^]^ Due to this fundamental incompatibility with the CMP process, precision planarization of Cu/polymer interface remained a challenge.

In this study, we propose a surface modification method using argon (Ar) plasma to enhance the CMP compatibility of BCB and thereby achieve reliable Cu/polymer hybrid bonding. The plasma treatment forms a hardened layer that prevents abrasive particle trapping and facilitates effective abrasion‐based material removal (Figure 1C). It also generates micro‐wrinkle structures with a hydrophilic surface that mitigate slurry flow restriction, therefore contributing to stable abrasion of the plasma‐modified BCB (p‐BCB) surface. We modeled the CMP mechanism in the p‐BCB/BCB bilayer and Cu hybrid structure to predict the dishing behavior, and as a result, successfully achieved a 230 nm BCB dishing structure within a wide process time window. Subsequently, we successfully achieved seamless hybrid bonding at 300°C using a conventional thermo‐compression process (Figure 1D), demonstrating its feasibility for advanced semiconductor packaging. The overall process flow of Ar plasma‐assisted CMP and hybrid bonding is summarized in Figure 1E, with detailed steps provided in Figure S1 (Supporting Information).

## 2 Results and Discussion

### 2.1 Ar Plasma Treatment for Surface Modification Favorable to CMP

For Cu/polymer hybrid bonding, it is essential to planarize the polymer in its half‐cured state, which is challenging due to its inherent ductility and flexibility. We propose a solution to this issue through Ar plasma surface treatment. As a polymer dielectric material, we used a divinylsiloxane‐benzocyclobutene (DVS‐BCB, hereafter referred to as BCB) thin film, which is widely used in both front‐end micro‐electro‐mechanical systems (MEMS) fabrication and back‐end processes such as redistribution layers (RDLs),^[^ ^45^ ^]^ owing to its excellent dielectric properties, thermal stability, and excellent mechanical reliability (Figure S2 and Table S1, Supporting Information).^[^ ^31^, ^46^, ^47^ ^]^

By optimizing the plasma treatment conditions, we found that the BCB's surface morphology was transformed into a micro‐wrinkle structure with enhanced mechanical hardness. These wrinkle structures act as local channels that facilitate slurry flow during the CMP process, thereby improving slurry distribution and suppressing hydroplaning to enhance polishing uniformity (Figure S3, Supporting Information).^[^ ^36^, ^48^ ^]^ This effect is particularly advantageous considering that the half‐cured BCB surfaces, due to their viscoelastic nature, hinder slurry delivery.

**Figure 2:**
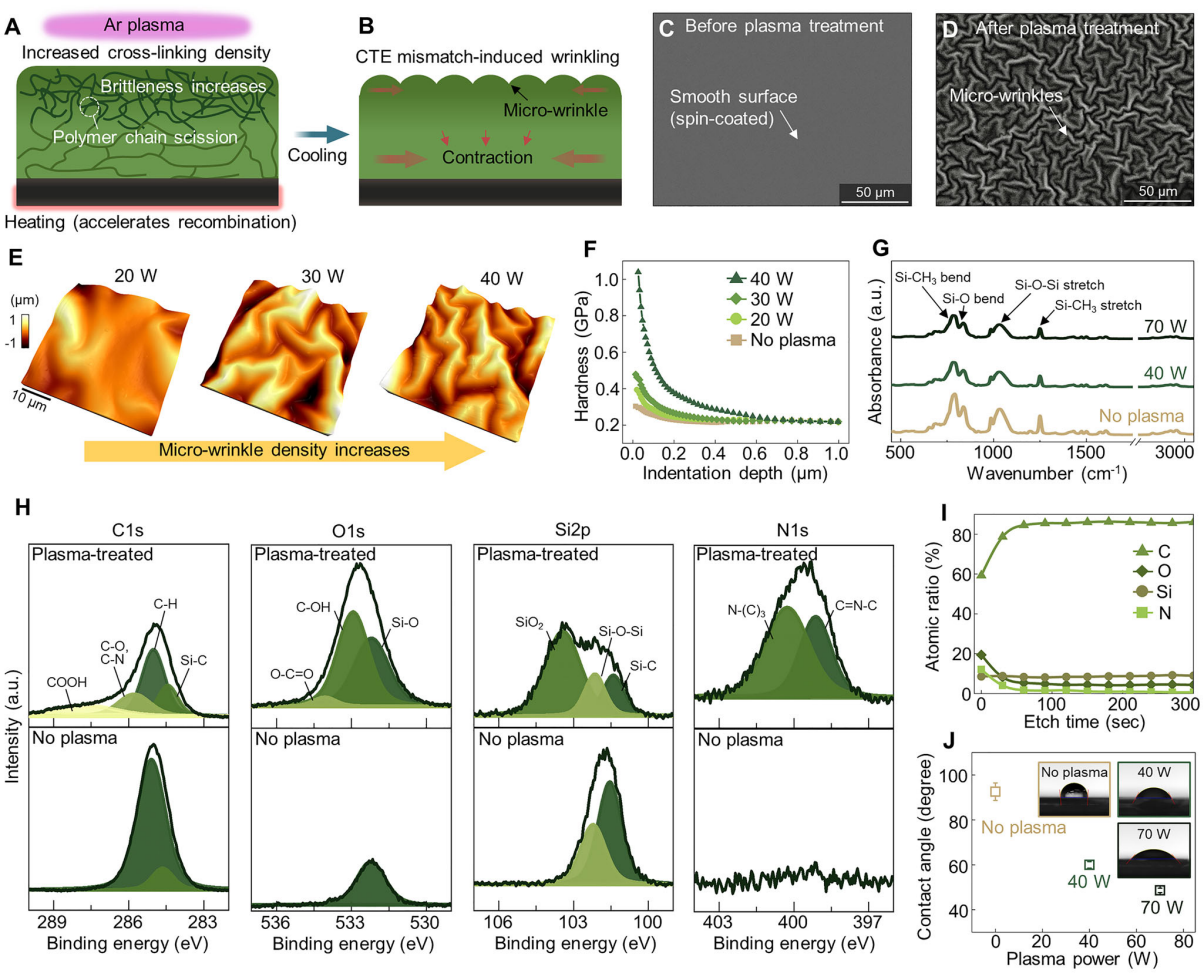
Manipulation of the morphology, mechanical, and chemical properties of DVS‐BCB films via Ar plasma. A) Schematic illustration of Ar plasma‐induced surface modification, showing polymer chain scission, enhanced radical recombination under heating, and increased crosslinking density near the surface. B) Formation of micro‐wrinkle structures due to the mismatch in the CTE between the p‐BCB surface and the compliant underneath. SEM images of half‐cured BCB surfaces C) before and D) after Ar plasma treatment. The untreated surface exhibits a smooth morphology as a result of spin‐coating, whereas p‐BCB shows the formation of micro‐wrinkles due to surface modification. E) AFM topography images of BCB surfaces treated with different plasma powers (20, 30, and 40 W), showing increased wrinkle density with higher power. F) Surface hardness of the BCB surface according to plasma power determined by nanoindentation. G) FTIR spectra show a gradual decrease in the absorbance of Si‐CH_3_ and Si‐O‐Si peaks via Ar plasma, indicating progressive bond scission. H) XPS spectra of C1s, O1s, Si2p, and N1s regions before and after plasma treatment, showing the formation of polar functional groups and the oxidation of siloxane to SiO_2_. I) XPS depth profile showing changes in atomic ratios after Ar plasma treatment, with increased oxygen and decreased carbon ratios near the surface. J) Contact angle measurements as a function of plasma power, demonstrating improved surface wettability via Ar plasma.

The surface deformation mechanism can be explained as follows. Ar plasma treatment was performed under optimized conditions (power: 40 W, working pressure: 2.9 × 10^−2^ Torr, treatment time: 30 s, temperature: 150 °C) to ensure an adequate surface modification thickness (Figures S4 and S5, Supporting Information). Under these conditions, the energetic Ar ions cleave the polymer chains, producing a high density of highly reactive free radicals.^[^ ^49^ ^]^ Elevated temperatures further enhance the mobility of polymer chains and increase the reactivity of generated radicals, thereby promoting crosslinking reactions.^[^ ^50^ ^]^ As a result, radical recombination actively occurs at the BCB surface, leading to a localized increase in crosslinking density and the formation of a hardened surface layer (**Figure** **2**A). Subsequently, due to the coefficient of thermal expansion (CTE) difference between the p‐BCB and underlying bulk BCB (the higher polymer chain density at p‐BCB lowers the CTE than that of BCB),^[^ ^51^ ^]^ the surface undergoes differential contraction upon cooling after plasma treatment. This mismatch limits the shrinkage of the stiffened surface layer relative to the more compliant underlying material, generating in‐plane compressive stress at the interface and forming periodic and irreversible micro‐wrinkle structures on the surface (Figure 2B–D).^[^ ^52^, ^53^ ^]^

The formation of micro‐wrinkles on the BCB surface becomes more pronounced with increasing plasma power, and this generation process can be confirmed by varying the plasma power during treatment. Atomic force microscopy (AFM) measurements of wrinkle morphology under varying plasma power levels revealed that with higher plasma power, intensified chain scission and enhanced crosslinking density led to a reduced wrinkle wavelength and increased wrinkle height (Figure 2E).

Furthermore, the Ar plasma treatment also hardens the mechanical properties of the BCB surface, thereby effectively preventing abrasive particle entrapment during CMP. We quantitatively verified the changes in surface hardness using nanoindentation (Figure 2F). The surface hardness was evaluated using a Berkovich indenter with an incremental depth of less than 1 µm. While the untreated BCB exhibited a low nano‐scale hardness of 0.3 GPa, p‐BCB (at 40 W) showed a hardness exceeding 1.0 GPa, with a clear trend of increasing hardness at higher plasma power. This behavior is attributed to more effective cleavage of polymer chains by higher‐energy Ar ions and enhanced radical recombination, resulting in a denser crosslinked surface structure.

Next, the chemical properties of the BCB surface were analyzed using Fourier transform infrared spectroscopy (FTIR) (Figure 2G) to further understand the origin of the observed structural and mechanical changes. The FTIR results showed that, with increasing plasma power, the absorbance peaks corresponding to siloxane (Si‐O‐Si) and methyl (Si‐CH_3_) groups gradually decreased, indicating progressive cleavage of these bonds within the BCB matrix by the plasma.

The X‐ray photoelectron spectroscopy (XPS) results further confirmed the changes in chemical bonding states on the BCB surface (Figure 2H). After plasma treatment, the radicals generated during the bond‐breaking process not only contributed to increased surface crosslinking density but also formed polar functional groups such as ─OH and ─COOH. Notably, the Si2p spectra revealed partial degradation of the siloxane (Si‐O‐Si) structure, accompanied by the emergence of new peaks corresponding to silicon dioxide (SiO_2_). The XPS depth profile revealed changes in the atomic ratio, confirming an increase in oxygen content and a decrease in carbon content, thereby providing further evidence of surface oxidation induced by plasma treatment (Figure 2I).^[^ ^54^, ^55^ ^]^

Moreover, the p‐BCB surface, which was initially hydrophobic due to the presence of methyl groups, became hydrophilic, a favorable condition for slurry infiltration during CMP. In addition to the chemical modification, the formation of wrinkle structures further enhanced surface wettability by increasing surface roughness (Figure S6, Supporting Information).^[^ ^56^ ^]^ The contact angle, which was initially ≈ 96° on BCB, gradually decreased with increasing plasma power, reaching 60° at 40 W and 49° at 70 W (Figure 2J).

In summary, p‐BCB exhibited increased surface hardness, along with brittle behavior and hydrophilic characteristics, featuring wrinkle structures. This mechanical transformation facilitated more effective abrasion by abrasives and enhanced material removal efficiency during CMP, thereby providing a favorable surface condition for mechanical planarization.

### 2.2 Facile Material Removal of p‐BCB Surfaces by Mechanical Abrasion

**Figure 3:**
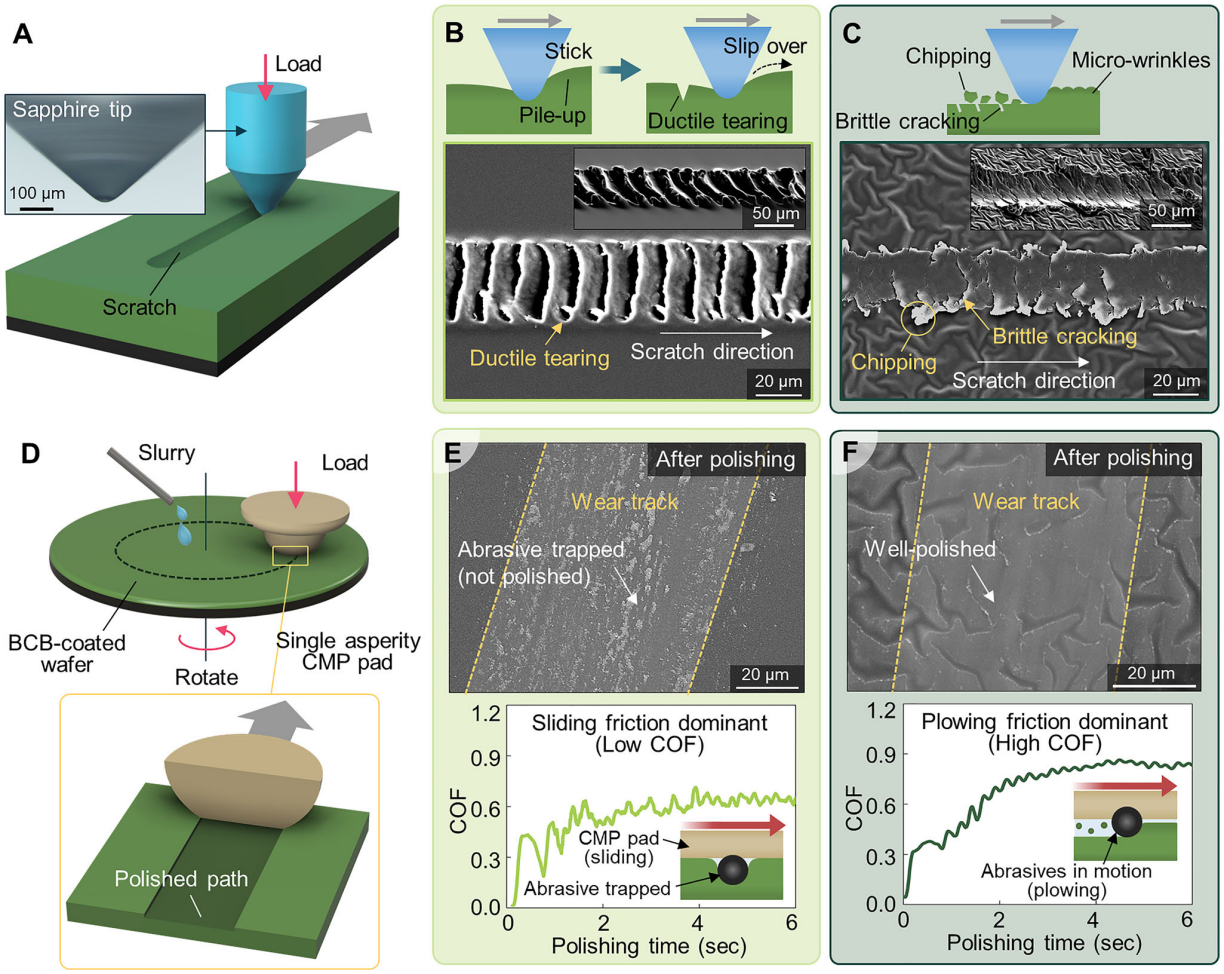
Enhanced material removal behavior of BCB surfaces after Ar plasma treatment. Schematics of A) the scratch test setup using a sapphire tip under constant normal load. B) Scratch mechanism and SEM image of untreated BCB, exhibiting ductile material behavior with periodic pile‐up and tearing patterns along the scratch direction. C) Scratch mechanism and SEM image of p‐BCB, showing brittle cracking and chipping behavior with reduced tip penetration due to surface hardening and micro‐wrinkle formation. D) Schematic illustration of the pin‐on‐disk experiment using a single pad asperity with CMP slurry. E) SEM image and COF profile of untreated BCB after polishing, showing abrasive particle trapping and a low COF associated with sliding‐dominant friction. F) SEM image and COF profile of p‐BCB after polishing, demonstrating clear material removal without abrasive trapping and a higher COF under plowing‐dominant friction with active abrasive polishing.

Next, we investigate the material removal behavior of BCB surfaces after the Ar plasma treatment. We first employed a scratch test to compare the deformation and wear characteristics of BCB films before and after the Ar plasma treatment (**Figure** **3**A). The experiment was conducted under a constant normal load using a sapphire tip with a blunt conical geometry and a radius of 50 µm. The resulting scratch tracks were analyzed to evaluate wear behavior, plastic deformation, and crack formation.

The half‐cured BCB surface before Ar plasma treatment exhibited ductile damage patterns, which are typical characteristics of soft polymers (Figure 3B).^[^ ^57^ ^]^ During the scratching process, repeated stick‐slip behavior was observed, in which the polymer in front of the moving tip was compressed and accumulated, while tensile stress built up behind the tip until it exceeded a critical threshold. The observed stick‐slip behavior caused ductile tearing of the surface, resulting in the formation of periodic tearing marks. These results indicate that BCB possesses both viscoelastic and ductile properties, and that ductile tearing is the dominant fracture mechanism. Such mechanical behavior is considered unfavorable for the CMP process.

In contrast, the p‐BCB surface showed distinct brittle fracture characteristics (Figure 3C). Under the same loading conditions, the width of the scratch track was narrower due to reduced tip penetration depth resulting from increased surface hardness. The scratch track exhibited irregular cracking and chipping along the inner region and sidewalls, where fractured fragments were detached from the surface. These features, namely brittle cracking and chipping, are indicative of a typical brittle material response, where stress is relieved primarily through crack propagation and material separation rather than plastic deformation.^[^ ^58^ ^]^ These observations suggest that Ar plasma treatment significantly reduced the ductility of the BCB surface while simultaneously increasing its hardness and brittleness.

We also performed an asperity‐scale pin‐on‐disk CMP test to quantitatively evaluate the material removal efficiency and frictional behavior of BCB under three‐body abrasion conditions, using a CMP pad with a single hemispherical asperity (Figure 3D). In this setup, a BCB‐coated wafer disk was rotated while the slurry was continuously supplied, and a constant normal load was applied using a CMP pad mounted on the pin. This configuration generated a circular polished track, allowing for detailed analysis of asperity‐scale friction and material removal behaviors.

For the half‐cured BCB, the polished track after the pin‐on‐disk experiment showed abrasives embedded in the surface without evidence of material removal, indicating that the particles were retained rather than engaged in effective abrasion (Figure 3E). This result suggests that the CMP pad slid over the polymer surface without effective abrasion, resulting in a sliding‐dominant friction regime. Consistently, the coefficient of friction (COF) measured during the process remained relatively low, ≈ 0.6.

In contrast, for the p‐BCB surface, the micro‐wrinkle structures served as channels to facilitate slurry flow. After the pin‐on‐disk experiment, a distinct wear track was observed where these wrinkle structures had been removed, indicating that polishing had occurred (Figure 3F). This suggests that under the applied load from the CMP pad asperity, the abrasive particles indented into the BCB surface and underwent relative motion, leading to actual material removal. Under this plowing‐dominant friction regime, the frictional force increased, and the COF rose to ≈ 0.9, further supporting the presence of a stable, abrasion‐driven polishing mechanism. As polishing progressed, the COF, which initially peaked at 1.03 during the abrasion of the p‐BCB surface, gradually decreased and stabilized, reaching a maximum of 0.71 with an average value of 0.64 (Figure S7, Supporting Information). Such reduction and convergence indicate that the p‐BCB layer was completely removed, exposing the underlying partially cured BCB surface. These COF variations provide a quantitative measure of the p‐BCB layer thickness and can be applied for endpoint detection in CMP processes (Figures S4, S5, and S8, Supporting Information).

### 2.3 BCB Dishing Control During CMP for Cu/BCB Hybrid Bonding

**Figure 4:**
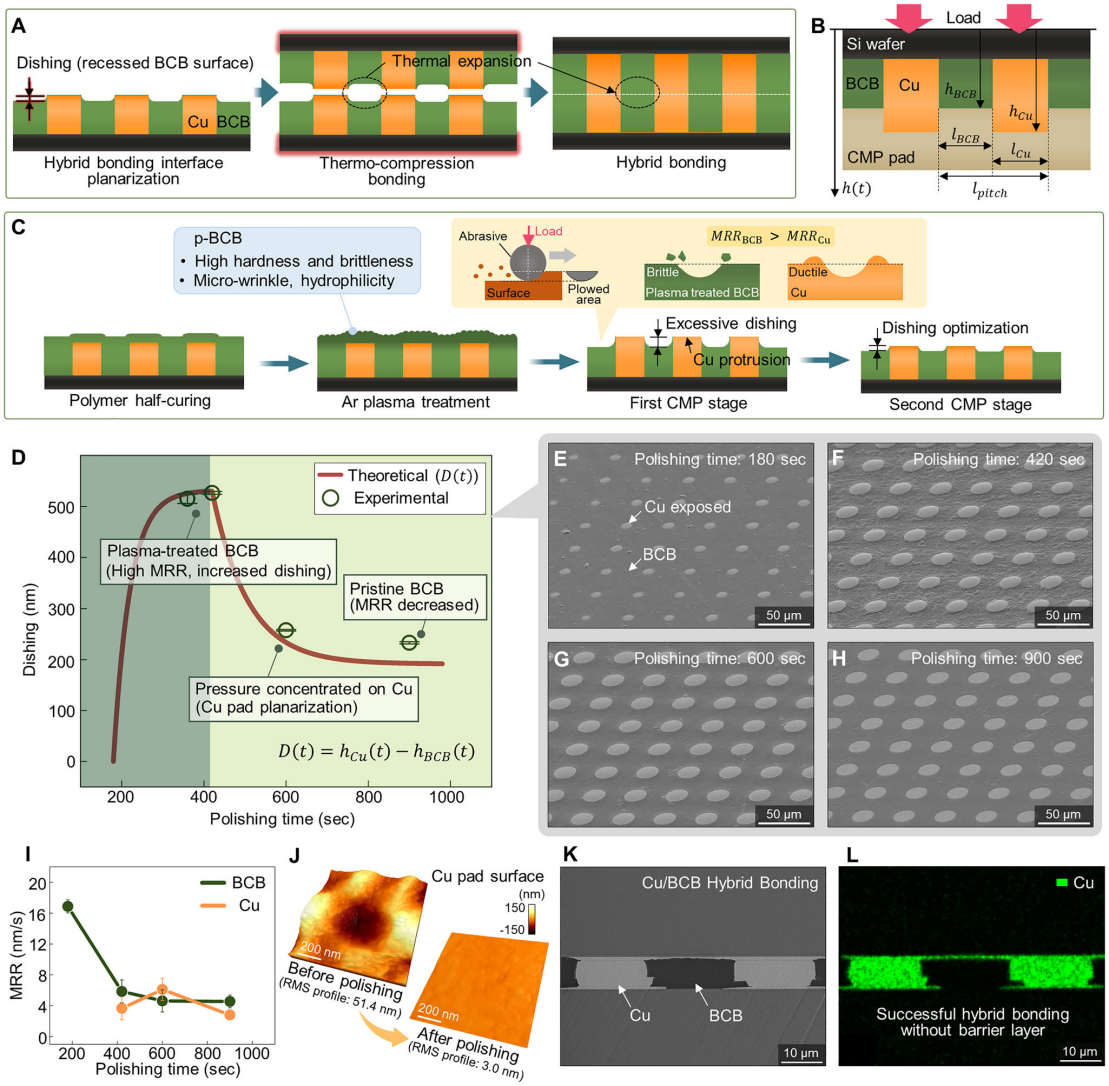
Ar plasma‐assisted CMP for hybrid bonding of Cu/BCB patterned wafers. A) Schematic illustration of Cu/BCB hybrid bonding. To compensate for the thermal expansion mismatch between Cu and BCB during bonding, the BCB surface is intentionally recessed. B) Cross‐sectional schematic illustrating pressure redistribution during CMP, governed by pad deformation and height differences between Cu and BCB regions. C) CMP process strategy for dishing depth control in Cu/BCB interfaces. During the first stage, faster removal of brittle p‐BCB causes Cu protrusion and localized pressure, driving gradual convergence of the dishing profile. D) Comparison of theoretical and experimental dishing depth over polishing time, capturing the transition from p‐BCB to untreated pristine BCB. E–H) SEM images showing surface evolution during polishing at different time intervals, (E) 180 s, (F) 420 s, (G) 600 s, and (H) 900 s. I) Measured MRR of BCB and Cu as a function of polishing time. (J) AFM images of the Cu surface before and after CMP showed a reduction in surface roughness from RMS 51.4 ± 9.1 to 3.0 ± 0.2 nm. K) Cross‐sectional SEM image of Cu/BCB interface showing successful hybrid bonding. L) EDS mapping result confirms the absence of Cu diffusion into the BCB layer, validating reliable bonding without the need for a diffusion barrier.

In hybrid bonding, BCB dishing must be precisely controlled based on factors such as the layout of metal and dielectric lines, including their width and pitch, as well as bonding conditions, including temperature and pressure.^[^ ^59^, ^60^ ^]^ At these temperatures, the difference in the coefficients of thermal expansion (CTE) between the materials must be considered during the CMP process to ensure a flat surface at the bonding temperature. In conventional Cu/SiO_2_ hybrid bonding, a dished Cu surface is required due to the higher CTE of Cu compared to SiO_2_, which helps to accommodate thermal mismatch. In contrast, for Cu/BCB hybrid bonding, BCB exhibits a greater CTE than Cu, necessitating a dished BCB surface to accommodate the thermal expansion mismatch (**Figure** **4**A). Moreover, to achieve reliable bonding, the p‐BCB surface layer must be completely removed.

To this end, we developed a mathematical dishing model based in part on previously proposed analytical frameworks for CMP dishing and step height reduction.^[^ ^61^ ^]^ The model considers the non‐uniform pressure distribution and the variation in material removal rate (MRR) between two surfaces during CMP. Here, MRR refers to the rate at which material is removed during the polishing process. In this model, we made several assumptions as follows. The CMP pad is modeled as an elastic body, and nominal contact pressure is assumed to act on both the Cu and BCB regions based on the surface height differences across the wafer. This pressure is proportional to the local elastic deformation of the pad, following Hooke's law. The total applied load is distributed between the Cu and BCB regions.

According to Preston's equation, the MRR in each region is proportional to the product of local pressure and relative velocity. The geometrical parameters are defined as follows (Figure 4B). Here, *T_pad_* denotes the pad thickness, and *h*(*t*) denotes the vertical displacement of the polishing pad surface at time *t*, resulting from cumulative material removal. The local surface heights of the Cu and BCB regions are denoted by *h_Cu_* and *h_BCB_*, respectively, and the widths (or area fractions) are denoted as *l_Cu_* and *l_BCB_*. More details on the model can be found in the Supplementary Text.

Based on the developed model, we propose a strategy to precisely control the dishing depth by selecting a suitable slurry and optimizing the CMP process. This strategy considers two different CMP stages (Figure 4C). In the first CMP stage of this model, although the surface hardness of p‐BCB is comparable to that of Cu (≈ 1.3 GPa), its significantly increased brittleness results in distinct polishing behavior. As a brittle material, p‐BCB is more susceptible to fracture and material removal under abrasive contact. In contrast, ductile Cu undergoes plastic deformation and pile‐up, resulting in a lower MRR despite its similar hardness. Consequently, p‐BCB exhibits a higher MRR than Cu during this stage.

In the second CMP stage, the rapid removal of p‐BCB results in a concentration of contact pressure on the protruding Cu regions. Considering the design of the test wafer and the CTE mismatch between Cu and BCB, it is necessary to control the BCB dishing depth to 230 nm (Figure S9 and Table S2, Supporting Information). To achieve this, the process conditions were optimized such that the MRR of Cu remained higher than that of BCB, and the degree of pressure concentration on the protruding Cu regions was quantitatively analyzed using the proposed model. To further promote Cu removal, a colloidal silica‐based slurry containing 1 wt.% hydrogen peroxide (H_2_O_2_) was employed. The H_2_O_2_ in the slurry promotes Cu removal by inducing surface oxidation, while showing no measurable chemical effect on BCB (Figures S10–S12, Supporting Information).^[^ ^62^, ^63^ ^]^ This selective mechanism, combined with pressure concentration, enables controlled CMP at the heterogeneous Cu/BCB interface. For process consistency, the same slurry was used from the first CMP stage. Under these conditions, the initial MRR of p‐BCB reached up to 17.3 nm/s and gradually decreased over time, whereas untreated BCB exhibited a much lower MRR of 3.1 nm s^−1^ (Figures 4I; S13, Supporting Information).

With the CMP conditions optimized based on the proposed model, the dishing behavior was evaluated (Figure 4D). The graph presents both the predicted dishing depth based on the proposed model (solid line) and the experimental values (open dots). The test wafer consisted of cylindrical Cu pads (20 µm diameter, 10.3 µm height) arranged with a 40 µm pitch (Figure S14, Supporting Information), and a BCB layer of 13.1 µm thickness was spin‐coated over the patterns. SEM images corresponding to CMP durations of 360, 420, 600, and 900 s are also presented (Figure 4E–H). At 180 s, p‐BCB was removed, and the Cu pads began to be exposed. From 180 to 420 s, the residual p‐BCB continued to be removed, resulting in a sharp increase in dishing depth. As the p‐BCB was completely removed, Cu protrusions exceeding 500 nm were observed, leading to a concentration of contact pressure on the elevated Cu regions and further shifting the mechanical load away from the recessed BCB areas. Between 420 and 600 s, this redistribution of pressure led to an increase in the Cu removal rate and accelerated planarization. As the Cu protrusion gradually diminished, the Cu MRR also slowly decreased. Eventually, beyond 600 s, a balance between the MRRs of Cu and BCB was achieved, resulting in a final BCB dishing depth of ≈ 230 nm.

To ensure void‐free and reliable hybrid bonding, the bonding interface must exhibit sufficiently low surface roughness. Half‐cured BCB inherently undergoes self‐planarization at bonding temperatures, enabling the formation of a smooth dielectric surface. Our results show that the surface roughness of Cu was reduced from an initial root mean square (RMS) value of 51.4 to 3.0 nm after CMP (Figure 4J), confirming the achievement of smooth surface conditions required for bonding.

Following CMP, two polished Cu/BCB patterned wafers were successfully bonded via thermo‐compression at 300 °C under vacuum, with no observable voids or interfacial delamination, and the bonded interfaces further exhibited excellent mechanical reliability (Figures 4K; S15 and S16, Supporting Information). Additional energy dispersive spectroscopy (EDS) analysis confirmed that no Cu diffusion occurred into the BCB layer, demonstrating that reliable hybrid bonding can be achieved without the use of a barrier layer (Figure 4L). The bonded structures also exhibited long‐term thermal reliability, as confirmed by thermal cycling tests (Figure S17, Supporting Information). These results validate that BCB dielectric planarization and hybrid bonding can fulfill the interface integrity and reliability requirements for heterogeneous integration systems.

## 3 Conclusion

In this study, we proposed and systematically validated an Ar plasma‐assisted surface modification technique to enable precision planarization of Cu/BCB hybrid bonding interfaces for advanced high‐density semiconductor packaging. Ar plasma treatment enhanced the surface hardness and brittleness of BCB, increased its surface hydrophilicity, and induced the formation of micro‐wrinkle structures. These combined effects collectively enabled high‐precision CMP of the BCB surface.

Furthermore, the p‐BCB/BCB bilayer structure incorporating Cu pads enabled precise control of dishing through an optimized CMP strategy. Through this approach, we achieved global planarization of the Cu/BCB patterned wafer, followed by successful hybrid bonding without voids or delamination, thereby demonstrating the reliability and process compatibility of the proposed method.

This work provides a technologically simple yet effective approach to processing viscoelastic, soft polymer dielectrics, addressing the challenges faced by conventional planarization methods. Notably, the proposed method meets key requirements for hybrid bonding, including uniform surface planarization and dishing values optimized for thermal expansion, making it suitable for a wide range of polymer‐based interconnect technologies. Therefore, the proposed method enhances both process compatibility and interfacial reliability in polymer‐based hybrid bonding, providing a practical foundation for next‐generation packaging platforms, such as polymer interposers and fine‐pitch interconnects.

## 4 Experimental Section

### Materials

The DVS‐BCB material used in this study was Cyclotene 3000, supplied by Dow Chemical, and the adhesion promoter AP3000 from the same company was used to enhance adhesion. Silicon wafers used for BCB spin‐coating and subsequent Ar plasma treatment were obtained from iNexus. Patterned wafers for Cu/BCB planarization experiments were provided by the National NanoFab Center in Korea, and 8‐inch wafers were diced into 8 mm × 8 mm chips for experimental use. The CMP process employed a colloidal silica‐based slurry (ACESOL‐2580, Ace Nanochem), to which 1 wt.% hydrogen peroxide (H_2_O_2_, Sigma–Aldrich) was added to promote copper removal, while a KONI pad (KPX Chemical) was used as the polishing pad. For the pin‐on‐disk experiments, a custom‐made polishing pad featuring a single hemispherical asperity 100 µm in diameter, fabricated from polyurethane resin (Smooth‐Cast 61D, Smooth‐On Inc.), was used.

### Sample Preparation

DVS‐BCB was deposited using a spin‐coating method with a spin coater (ACE‐200, Dong AH Trade Corp). Before coating, an adhesion promoter (AP3000) was applied to enhance adhesion between the SiO_2_ surface and the BCB layer, using spin speeds of 500 rpm for 5 s and 3000 rpm for 30 s. The BCB layer was then coated using the same equipment at 500 rpm for 10 s followed by 1200 rpm for 60 s. After coating, the wafer was baked on a hot plate (MSH‐20D, DAIHAN Scientific) at 130 °C for 80 s to remove the mesitylene solvent. Subsequently, a B‐stage curing process was performed in a box furnace (AWF 12/12, Lenton) at 220 °C for 15 min.

Plasma surface treatment was performed using an Ar plasma system (Atomic‐Premium, CN1) under the following conditions: temperature of 150 °C, working pressure of 2.9 × 10‐2 Torr, Ar gas flow rate of 100 sccm, plasma power ranging from 20 to 70 W, and a treatment duration of 30 s. For scratch tests and the fabrication of Cu/BCB patterned wafers, plasma treatment was conducted at 40 W for 30 s.

### Mechanical Testing

Nanoindentation was performed using an iNano nanoindenter (KLA Instruments) equipped with a Berkovich diamond tip to measure the surface hardness of BCB films. For scratch testing, a blunt conical sapphire tip with a 50 µm radius and a 45° attack angle was used with the UMT TriboLab (Bruker). The tests were conducted at a constant load of 50 mN with a sliding speed of 0.05 mm/s. A unidirectional sliding motion was applied over a 10 mm distance, and each test was repeated three times at different positions to ensure reproducibility.

### Polishing and Bonding Experiments

Pin‐on‐disk experiments were performed using a UMT TriboLab system (Bruker) to evaluate the frictional behavior and material removal characteristics on BCB surfaces. The tests were conducted under a normal load of 20 mN at a radial distance of 15 mm from the center of rotation, with a rotational speed of 255 rpm, corresponding to a linear sliding speed of ≈ 0.4 m s^−1^. During the tests, the slurry was continuously supplied.

CMP of the Cu/BCB patterned wafers was performed using a MultiPrep^TM^ System‐8 polisher (Allied High Tech Products). Both the CMP pad and the carrier head were rotated at 30 rpm under an applied pressure of 46 kPa, and the slurry was continuously supplied at a flow rate of 100 ml/min. The MRR was calculated based on changes in the thickness of BCB and Cu pads measured at different polishing times. Polishing experiments were conducted at 0, 180, 420, 600, and 900 s, and for each condition, dishing depth was measured, and cross‐sectional analysis was performed to determine the thickness of each layer (*n* = 3, where n is the number of experiments conducted for generating statistical data).

Hybrid bonding was carried out using a thermo‐compression bonder (FC150, SUSS MicroTec) under vacuum at a nominal bonding pressure of 1.07 MPa (Figure S15, Supporting Information). The bonding process consisted of a 200 s ramp‐up stage, a 300 s holding stage at 300 °C, and 500 s cooling stage, during which the pressure was kept constant.

### Surface and Structural Characterization

AFM measurements (NX10, Park Systems) were used to analyze surface wrinkle morphology, roughness, and dishing, with a scan resolution of 256 × 256 pixels. Each measurement was repeated three times (*n* = 3) for statistical analysis. FTIR spectra were acquired using a Nicolet iS50 system (Thermo Fisher Scientific) to track chemical changes in the BCB film. Contact angle measurements were performed using a contact angle analyzer (SEO Phoenix) to evaluate surface wettability. XPS analysis was conducted using the Axis‐Supra system (Kratos Analytical) to identify elemental composition and bonding states. SEM images were obtained using an SU5000 microscope (Hitachi), and for cross‐sectional analysis, ion milling was performed using an ArBlade5000 system (Hitachi). EDS elemental mapping was conducted using the QUANTAX EDS system (Bruker) to confirm the absence of Cu diffusion, with maps acquired at a resolution of 512 × 400 pixels, a dwell time of 50 µs per pixel, and 32 scans with summation averaging to ensure reliable signal quality.

## Conflict of Interest

The authors declare no conflict of interest.

## Supporting information



Supporting Information

## Data Availability

The data that support the findings of this study are available from the corresponding author upon reasonable request.

## References

[1] J. U. Knickerbocker , R. Budd , B. Dang , Q. Chen , E. Colgan , L.‐W. Hung , S. Kumar , K. W. Lee , M. Lu , J. W. Nah , R. Narayanan , K. Sakuma , V. Siu , B. Wen , In *Proceedings of the 2018 IEEE 68th Electronic Components and Technology Conference* (ECTC), IEEE, Piscataway, NJ 2018, 1519–1528.

[2] Z. Chen , J. Zhang , S. Wang , C.‐P. Wong , Fundam. Res. 2024, 4, 1455. 39734548 10.1016/j.fmre.2023.04.014 PMC11670716

[3] J. Ryckaert , S. B. Samavedam , Nat. Rev. Electr. Eng. 2024, 1, 139.

[4] Z.‐H. Li , T.‐C. Chiang , P.‐Y. Kuo , C.‐H. Tu , Y. Kuo , P.‐T. Liu , Adv. Sci. 2023, 10, 2205481. 10.1002/advs.202205481 PMC10037976 36658711

[5] J. H. Lau , IEEE Trans. Compon., Packag., Manuf. Technol. 2022, 12, 228.

[6] C. Choi , H. Kim , J.‐H. Kang , M.‐K. Song , H. Yeon , C. S. Chang , J. M. Suh , J. Shin , K. Lu , B.‐I. Park , Y. Kim , H. E. Lee , D. Lee , J. Lee , I. Jang , S. Pang , K. Ryu , S.‐H. Bae , Y. Nie , H. S. Kum , M.‐C. Park , S. Lee , H.‐J. Kim , H. Wu , P. Lin , J. Kim , Nat. Electron. 2022, 5, 386.

[7] Z.‐J. Hong , D. Liu , H.‐W. Hu , C.‐K. Hsiung , C.‐I. Cho , C.‐H. Chen , J.‐H. Liu , M.‐W. Weng , M.‐P. Hsu , Y.‐C. Hung , K.‐N. Chen , Appl. Surf. Sci. 2023, 610, 155470.

[8] C. Jeon , S. Kang , M. E. Kim , J. Park , D. Kim , S. Kim , K. M. Kim , ACS Appl. Mater. Interfaces. 2024, 16, 48481. 39190606 10.1021/acsami.4c08390

[9] Y.‐C. Huang , Y.‐X. Lin , C.‐K. Hsiung , T.‐H. Hung , K.‐N. Chen , Nanomaterials 2023, 13, 2490.37687000

[10] J. Choi , S. Jang , D. Lee , S. Kang , S. Kim , S. E. Kim , J. Semiconductor Technol. Sci. 2025, 25, 102.

[11] J. A. Theil , L. Mirkarimi , G. Fountain , G. Gao , R. Katkar , In Proceedings of the 2019 International Wafer Level Packaging Conference (IWLPC), IEEE, Piscataway, NJ 2019, 1–6.

[12] H.‐W. Hu , K.‐N. Chen , Microelectron. Reliab. 2021, 127, 114412.

[13] K.‐N. Chen , Z. Xu , J.‐Q. Lu , IEEE Electron Device Lett. 2011, 32, 1119.

[14] S. Moreau , J. Jourdon , S. Lhostis , D. Bouchu , B. Ayoub , L. Arnaud , H. Fremont , ECS J. Solid State Sci. Technol. 2022, 11, 024001.

[15] J. Utsumi , K. Ide , Y. Ichiyanagi , Micro. Nano. Eng. 2019, 2, 1.

[16] Q. Kang , C. Wang , S. Zhou , G. Li , T. Lu , Y. Tian , P. He , ACS Appl. Mater. Interfaces. 2021, 13, 38866. 34318673 10.1021/acsami.1c09796

[17] F. Niu , X. Wang , S. Yang , S. Xu , Y. Zhang , T. Suga , C. Wang , Appl. Surf. Sci. 2024, 648, 159074.

[18] X.‐B. Le , S.‐H. Choa , Micromachines 2024, 15, 1332.39597143

[19] J. Fuse , Y. Yoshihara , M. Sano , F. Inoue , In Proceedings of the 2024 IEEE 10th Electronics System‐Integration Technology Conference (ESTC) , IEEE, Piscataway, NJ 2024, 1–5.

[20] A. Chacko , D. Bai , R. Puligadda , In Direct Copper Interconnection for Advanced Semiconductor Technology, CRC Press, Boca Raton, FL, USA 2024, pp. 116–130.

[21] T. Tabata , L. Sanchez , V. Larrey , F. Fournel , H. Moriceau , Microelectron. Reliab. 2020, 107, 113589.

[22] I. Panchenko , L. Wenzel , M. Mueller , C. Rudolph , A. Hanisch , J. M. Wolf , IEEE Trans. Compon., Packag., Manuf. Technol. 2022, 12, 410.

[23] V. K. Khanna , J. Phys. D, Appl. Phys. 2010, 44, 034004.

[24] T. Workman , L. Mirkarimi , J. Theil , G. Fountain , K. M. Bang , B. Lee , C. Uzoh , D. Suwito , G. Gao , P. Mrozek , In Proceedings of the 2021 IEEE 71st Electronic Components and Technology Conference (ECTC) , IEEE, Piscataway, NJ 2021, 2071–2077.

[25] V. Dubey , D. Wünsch , K. Gottfried , M. Wiemer , T. Fischer , A. Hanisch , S. Schermer , C. Helke , M. Hasse , D. Reuter , S. E. Schulz , S. Ghosal , L. Hofmann , ECS Trans. 2023, 112, 73.

[26] A. U. Alam , M. M. R. Howlader , M. J. Deen , ECS J. Solid State Sci. Technol. 2013, 2, P515.

[27] H. Park , H. Seo , S. E. Kim , Electron. Mater. Lett. 2021, 17, 392.

[28] W. Kim , S. Choi , S. Lee , Y.‐C. Joo , B.‐J. Kim , Electron. Mater. Lett. 2025, 21, 184.

[29] J. H. Lau , Flip Chip, Hybrid Bonding, Fan‐In, and Fan‐Out Technology, Springer, Berlin, New York 2024.

[30] C. Netzband , D. Burns , K. Tapily , I. Son , C. Wajda , In Proceedings of the 2023 IEEE 73rd Electronic Components and Technology Conference (ECTC) , IEEE, Piscataway, NJ 2023, 350–355.

[31] S. P. Phansalkar , Y.‐H. Yang , C. Kim , B. Han , Y. K. Jee , C. S. Lee , U. Byung Kang , J. H. Lee , S. Cheon , In Proceedings of the 2021 IEEE 71st Electronic Components and Technology Conference (ECTC) , IEEE, Piscataway, NJ 2021, 1773–1778.

[32] P.‐S. He , C.‐W. Tu , K.‐C. Shie , C.‐Y. Liu , H.‐Y. Tsai , D.‐P. Tran , C. Chen , J. Electroanal. Chem. 2024, 969, 118544.

[33] C. Wang , H. Hao , K. Tajima , Adv. Sci. 2022, 9, 2201045. 10.1002/advs.202201045 PMC9165494 35347899

[34] C.‐K. Hsu , P.‐Y. Lin , W.‐Y. Lin , M.‐C. Yew , S.‐S. Yeh , K.‐C. Lee , J.‐H. Wang , P.‐C. Lai , C.‐C. Yang , S.‐P. Jeng , In Proceedings of the 2017 12th International Microsystems, Packaging, Assembly and Circuits Technology Conference (IMPACT) , IEEE, Piscataway, NJ 2017, 202–205.

[35] S. K. Tippabhotla , L. Ji , Y. Han , In Proceedings of the 2022 IEEE 72nd Electronic Components and Technology Conference (ECTC) , IEEE, Piscataway, NJ 2022, 1695–1703.

[36] J. Park , S. Kang , M. E. Kim , N. J. Kim , J. Kim , S. Kim , K. M. Kim , Adv. Mater. Technol. 2023, 8, 2202134.

[37] S. L. Lin , W. C. Huang , C. T. Ko , K.‐N. Chen , Microelectron. Reliab. 2012, 52, 352.

[38] X. Wang , F. Niklaus , In 3D and Circuit Integration of MEMS, Wiley, Hoboken, NJ, USA 2021, 331–359.

[39] P.‐S. He , D.‐P. Tran , K.‐C. Shie , C. Chen , Appl. Surf. Sci. 2025, 685, 162023.

[40] J. J. McMahon , F. Niklaus , R. J. Kumar , J. Yu , J. Q. Lu , R. J. Gutmann , MRS Online Proc. Libr. (OPL) 2005, 867, W4.

[41] S. Kim , N. Saka , J.‐H. Chun , IEEE Trans. Semiconductor Manuf. 2014, 27, 431.

[42] H. J. Ryu , D. G. Kim , S. Kang , J.‐h. Jeong , S. Kim , CIRP Ann. 2021, 70, 273.

[43] T. Chakraborty , P. Goradia , S. Verhaverbeke , H.‐W. Chen , C. Buch , P. Lianto , In Proceedings of the 2020 IEEE 22nd Electronics Packaging Technology Conference (EPTC) , IEEE, Piscataway, NJ 2020, 41–43.

[44] S. Jang , S. Lee , S. Park , S. E. Kim , In Proceedings of the 2024 International Conference on Electronics Packaging (ICEP) , IEEE, Piscataway, NJ 2024, 307–308.

[45] Y. Kwon , A. Jindal , R. Augur , J. Seok , T. S. Cale , R. J. Gutmann , J.‐Q. Lu , J. Electrochem. Soc. 2008, 155, H280.

[46] K. Sahoo , V. Harish , H. Ren , S. S. Iyer , IEEE Electron. Dev. Rev. 2024, 2, 6.

[47] P. Nimbalkar , P. Bhaskar , M. Kathaperumal , M. Swaminathan , R. R. Tummala , Polymers 2023, 15, 3895. 37835944 10.3390/polym15193895 PMC10575375

[48] L. J. Borucki , T. Sun , Y. Zhuang , D. Slutz , A. Philipossian , MRS Online Proc. Libr. 2009, 1157, 102.

[49] S. Ershov , F. Khelifa , V. Lemaur , J. Cornil , D. Cossement , Y. Habibi , P. Dubois , R. Snyders , ACS Appl. Mater. Interfaces. 2014, 6, 12395. 24979702 10.1021/am502255p

[50] E. M. Liston , L. Martinu , M. R. Wertheimer , J. Adhes. Sci. Technol. 1993, 7, 1091.

[51] G. Schmidt , Physics of High Temperature Plasmas, Elsevier, Amsterdam, New York 2012.

[52] M. Jalali‐Mousavi , S. K. S. Cheng , J. Sheng , Nanomaterials 2023, 13, 1044. 36985941 10.3390/nano13061044 PMC10054355

[53] Y. Tokudome , K. Suzuki , T. Kitanaga , M. Takahashi , Sci. Rep. 2012, 2, 683. 23002424 10.1038/srep00683 PMC3448069

[54] K. W. Paik , R. J. Saia , J. J. Chera , MRS Online Proc. Libr. 1990, 203, 303.

[55] K. W. Paik , H. S. Cole , R. J. Saia , J. J. Chera , J. Adhes. Sci. Technol. 1993, 7, 403.

[56] Z. Li , P. Liu , S. Chen , X. Liu , Y. Yu , T. Li , Y. Wan , N. Tang , Y. Liu , Y. Gu , Eur. Polym. J. 2023, 190, 111997.

[57] H. Jiang , R. Browning , H.‐J. Sue , Polymer 2009, 50, 4056.

[58] A. M. Kovalchenko , S. Goel , I. M. Zakiev , E. A. Pashchenko , R. Al‐Sayegh , J. Mater. Res. Technol. 2019, 8, 703.

[59] S. Park , S. Kang , D. G. Kim , S. Kim , CIRP Ann. 2025, 74, 465.

[60] H. H. Chu , D. H. Kim , J. H. Park , S. Kang , J. Son , H. Lee , H. Yoo , S.‐W. Kim , S. Kim , Y.‐J. Kim , Appl. Surf. Sci. 2025, 638, 162466.

[61] G. Fu , A. Chandra , IEEE Trans. Semiconductor Manuf. 2003, 16, 477.

[62] T. Du , D. Tamboli , V. Desai , S. Seal , J. Electrochem. Soc. 2004, 151, G230.

[63] D. Liu , Z. Zhang , J. Feng , Z. Yu , F. Meng , C. Shi , G. Xu , S. Shi , W. Liu , Colloids Surf. A 2023, 656, 130500.

