# Supplementary material for: Chemical Mechanical Polishing of Plasma‐Modified Cu/Polymer Interfaces for Advanced Hybrid Bonding
Source: Adv Sci (Weinh). 2025 Oct 29;13(3):e12611. doi: 10.1002/advs.202512611 (PMC12806195; doi:10.1002/advs.202512611)
Supplement: Supplementary file 1 — Supporting Information [file ADVS-13-e12611-s001.pdf]

Supporting Information

Chemical Mechanical Polishing of Plasma-Modified Cu/Polymer Interfaces for Advanced Hybrid Bonding

Sukkyung Kang, Chansu Jeon, Juseong Park, Seulah Park, Kyung Min Kim, Sanha Kim**

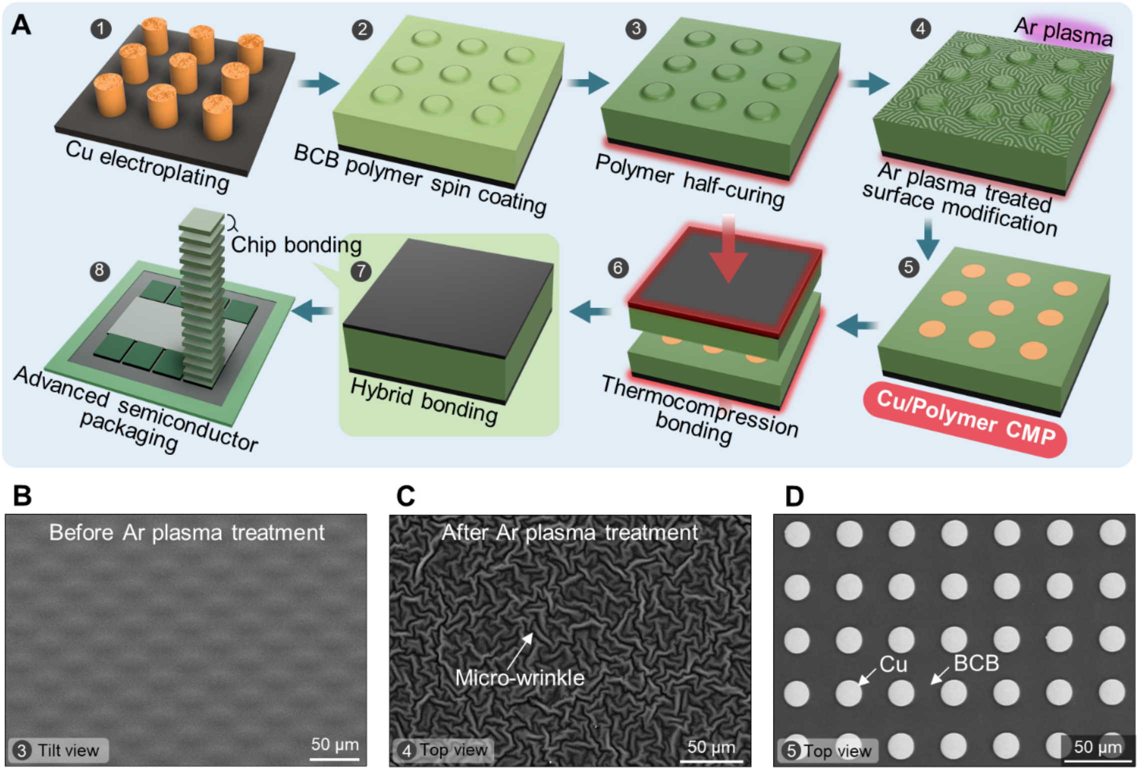

Figure S1. Process flow and surface modification in Ar plasma-assisted CMP for Cu/BCB hybrid bonding. (A) The process flow of Cu/polymer hybrid bonding, including Cu electroplating, BCB spin-coating, half-curing, Ar plasma surface modification, CMP, and thermo-compression bonding for advanced packaging. SEM images of (B) BCB polymer spin-coated on Cu-patterned wafer, (C) Ar plasma-modified BCB (p-BCB) surface showing the micro-wrinkle structures, (D) Cu/BCB interface after plasma-assisted CMP exhibiting a well-polished surface

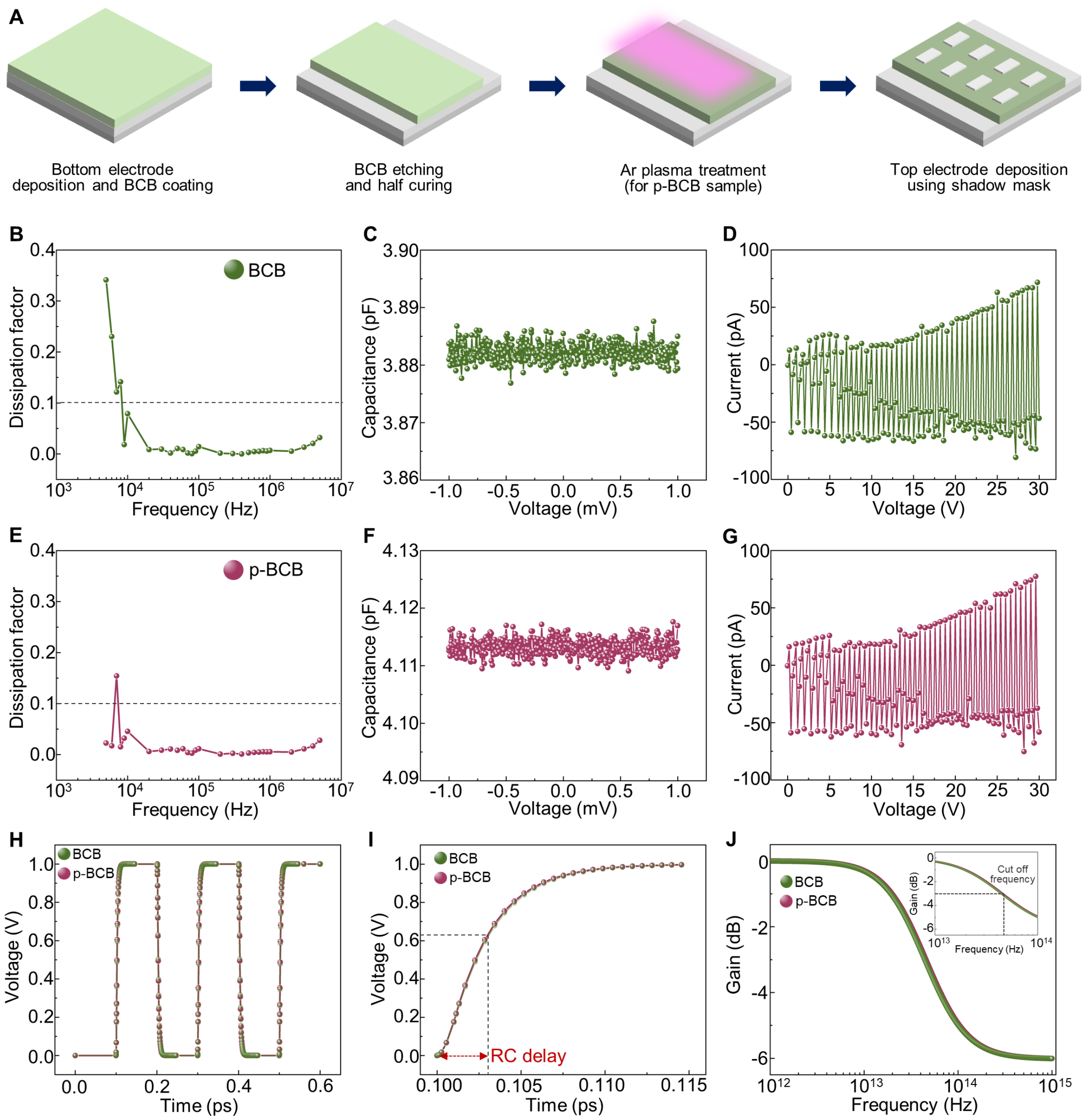

Figure S2. Electrical characterization of BCB and p-BCB using metal-insulator-metal (MIM) capacitor structures. (A) Schematic illustration of the fabrication process. First, a 60-nm-thick platinum (Pt) bottom electrode was deposited by e-beam evaporation. Subsequently, BCB films were spin-coated and half-cured, while for the p-BCB samples, an additional Ar plasma treatment was carried out. The coating, half-curing, and plasma treatment conditions were identical to those described in the main text. Finally, a 60-nm-thick Pt top electrode with an area of 2 × 1 mm^2 was deposited to complete the MIM capacitor structure. (B, E) Frequency-dependent dissipation factor (DF) of BCB and p-BCB, respectively. Both materials maintained DF values below 0.1 in the frequency range of 10 kHz to 5 MHz, demonstrating stable and reliable dielectric behavior. (C, F) Capacitance-voltage (C-V) characteristics measured at 1 MHz, where DF values remained below 0.1. The C-V response of both samples was

consistently stable, with capacitance values of 3.88 pF for BCB and 4.11 pF for p-BCB. The slightly higher capacitance of p-BCB is attributed to thickness variations caused by the wrinkled surface morphology and surface layer oxidation generated by the plasma treatment. (D, G) Current-voltage (I-V) characteristics measured under applied bias up to 30 V. No dielectric breakdown was observed in either material, and the leakage currents remained at very low levels of several tens of picoamperes, confirming excellent dielectric reliability for both BCB and p-BCB. (H, I) Transient response simulations using LTspice circuit models. Both BCB and p-BCB exhibited nearly identical RC delay behavior, and the extracted delay times revealed no significant differences between the two dielectrics. (J) Frequency response comparison of BCB and p-BCB derived from the circuit simulations. Both materials exhibited cut off frequencies above 10^{13} Hz, confirming their capability to support high-speed signal transmission without crosstalk between adjacent interconnect lines.

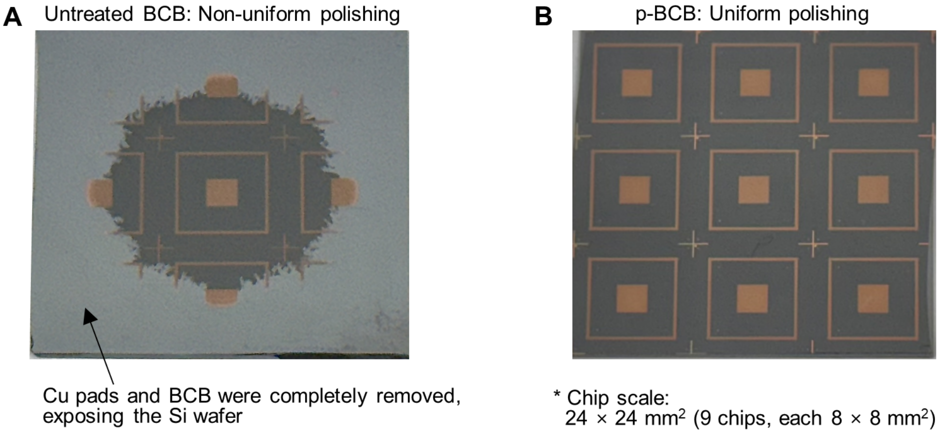

Figure S3. Comparison of polishing uniformity between untreated BCB and p-BCB. (A) Untreated BCB shows severe non-uniformity when polishing a 3 × 3 array of chips (9 chips, each 8 × 8 mm^2, total 24 × 24 mm^2). After polishing for 2000 s, the Cu pad located at the center of the middle chip, which had been covered by the BCB layer, was exposed as the BCB was removed. By that time, the Cu pads and BCB layers on the outer chips had already been completely removed, revealing the underlying Si wafer. This non-uniform removal was caused by both pressure imbalance and the viscoelastic nature of the polymer, which hindered slurry distribution to the center chip and concentrated polishing at the edges. The non-uniformity effect became even more pronounced compared to single-chip polishing because the slurry was not evenly distributed across the larger wafer-scale area. (B) p-BCB exhibited uniform polishing. The Cu in the central chip was exposed after only about 60 s of polishing, and the brittle p-BCB surface enabled effective slurry spreading and even material removal across the entire wafer. No excessive edge removal or loss of the BCB layer was observed. These results confirm that plasma-assisted CMP significantly improves both polishing efficiency and uniformity. All polishing experiments were carried out using POLI-400 equipment (GNP Technology) under conditions of 76.4 kPa and 90 rpm, using the same slurry and CMP pad as employed in the main experiments.

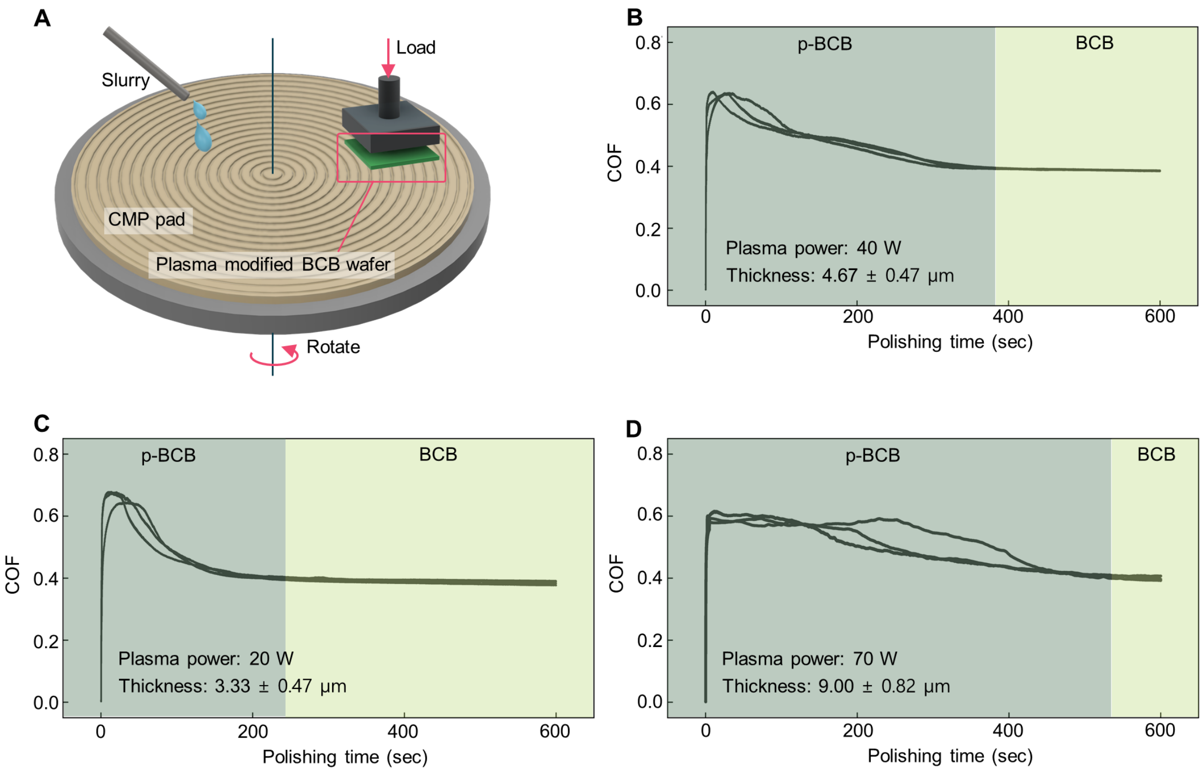

Figure S4. Quantitative evaluation of the p-BCB layer thickness using a pin-on-disk test. (A) Schematic of the pin-on-disk setup, where the pin specimen was a wafer chip coated with plasma-treated BCB, and the rotating plate was a CMP pad (KONI pad, KPX Chemical). The experiment was conducted using a UMT TriboLab system (Bruker), and the applied pressure and relative velocity were set identical to those used in the CMP process. At the initial stage of polishing, removal of the p-BCB layer resulted in a high COF, which gradually decreased and stabilized as the underlying pristine BCB was exposed. Based on the material removed until the COF reached the stabilized region, the thickness of the p-BCB layer was estimated as (B) 4.67 ± 0.47 μm at 40 W, (C) 3.33 ± 0.47 μm at 20 W, and (D) 9.00 ± 0.82 μm at 70 W (n = 3, where n is the number of experiments conducted for generating statistical data). In this study, a p-BCB thickness of 4.67 μm obtained at 40 W plasma treatment was applied, considering both the spin-coated BCB thickness above the Cu pads (approximately 2.8 μm) and the additional removal depth required for planarization after Cu exposure.

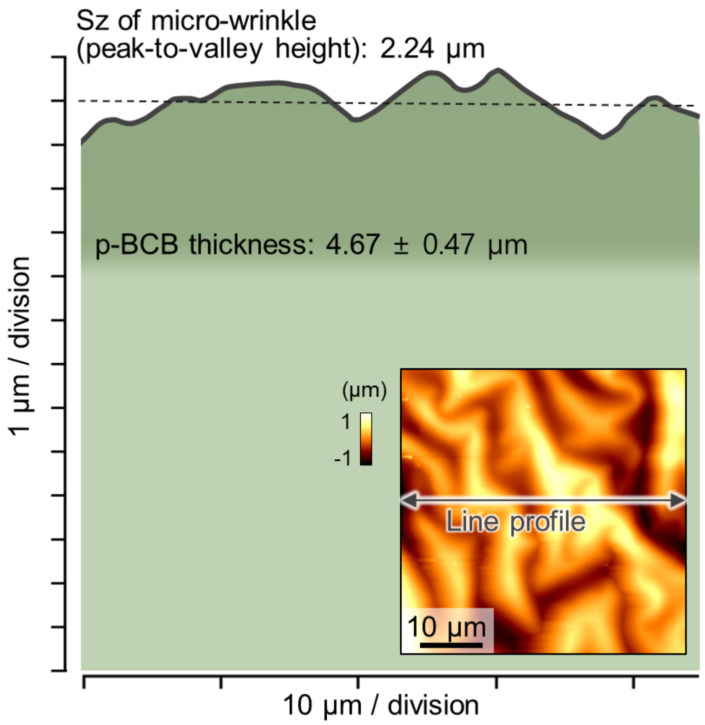

Figure S5. Schematic cross-sectional view considering the AFM-observed wrinkle structures and the thickness of the p-BCB surface. The peak-to-valley height (Sz) of the wrinkle structures is 2.24 μm, which is smaller than the p-BCB thickness (4.67 ± 0.47 μm), indicating that the modified layer extends beneath the wrinkle structures.

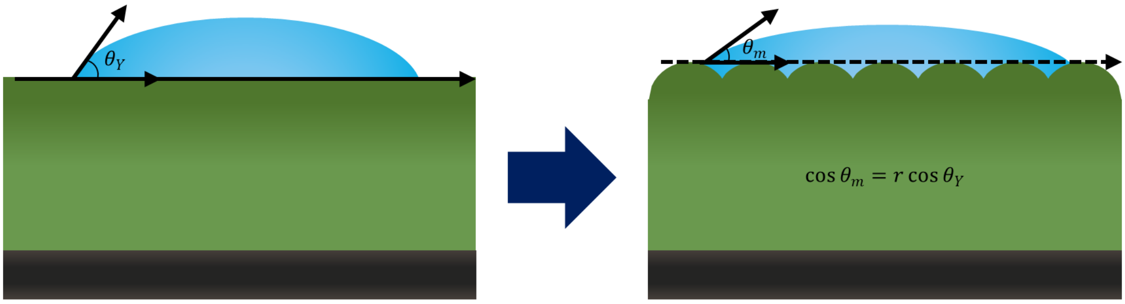

Figure S6. Enhancement of BCB surface wettability through Ar plasma-induced wrinkle structures. Plasma treatment introduces hydrophilic functional groups and forms microscale wrinkle structures, leading to improved wettability and increased surface roughness. According to Wenzel's model, surface roughness amplifies intrinsic wettability, lowering the apparent contact angle (θ_m) on an already hydrophilic surface. This is described by the equation:

$$\cos\theta_m = r\cos\theta_Y$$

θ_Y: Young's contact angle on a smooth surface

θ_m: apparent contact angle observed on a rough surface

r: roughness factor, defined as the ratio of actual to projected surface area ($r \geq 1$)

This dual chemical and topographical modification promotes uniform slurry spreading and effective infiltration, facilitating more efficient CMP.

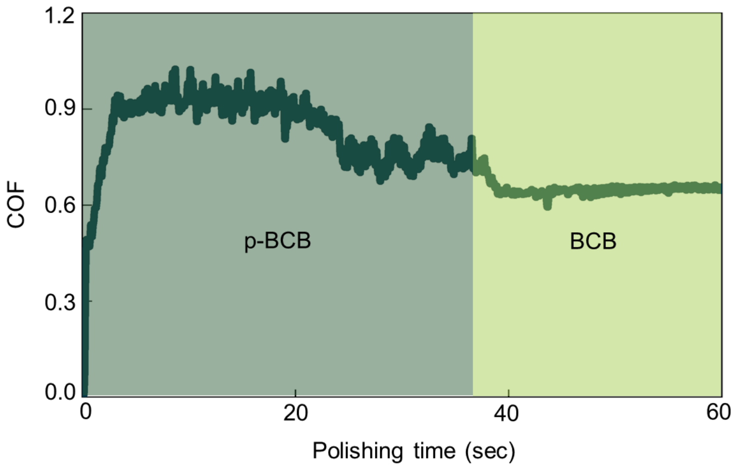

Figure S7. Time-dependent COF profile showing a transition from high-friction (p-BCB region) to low-friction (pristine BCB region) as polishing proceeds.

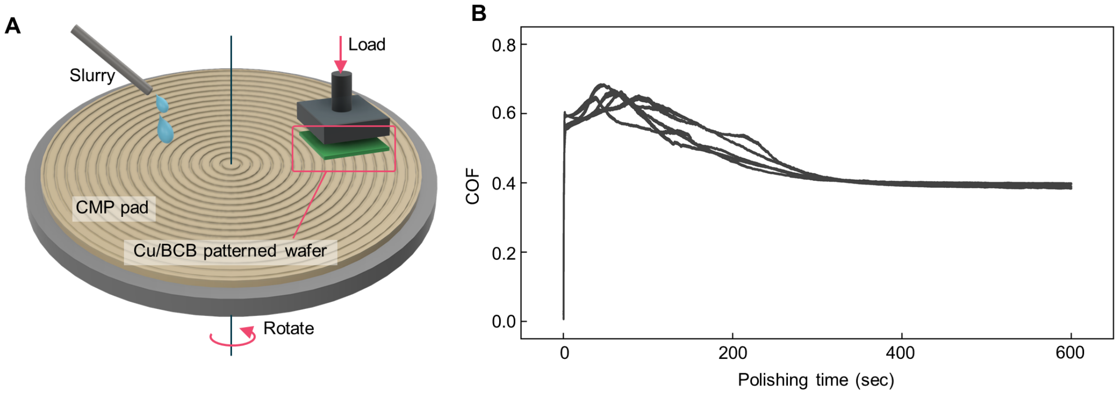

Figure S8. COF measurement based on pin-on-disk experiments. (A) Schematic of the pin-on-disk test configuration. The rotating disk was covered with a CMP pad, while the stationary pin was mounted with a Cu/BCB patterned wafer. The experiments were conducted under pressure and relative velocity conditions equivalent to those in CMP tests, and the COF was measured. (B) Variation of COF as a function of polishing time obtained from pin-on-disk experiments. During the removal of p-BCB, the COF increased up to 0.7 and subsequently converged to 0.4 as the pristine BCB layer was exposed. Based on these results, in situ COF monitoring during polishing could also be applied as a potential endpoint detection technique.

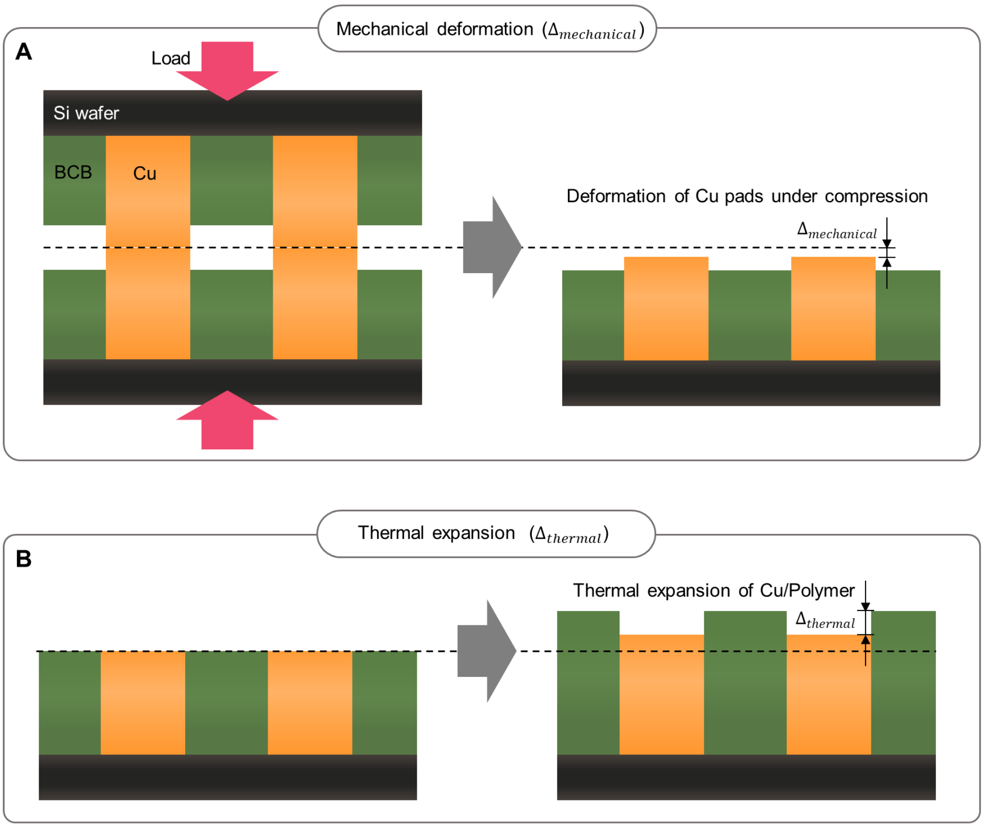

Figure S9. Schematic illustration of the calculation method for the appropriate dishing depth used in thermo-compression bonding. (A) Mechanical deformation ($\Delta_{mechanical}$): after planarization, Cu pads protrude above the polymer (BCB) surface, and the applied load is concentrated on the Cu, resulting in compressive deformation of the Cu pads. (B) Thermal expansion ($\Delta_{thermal}$): An additional height change occurs at the bonding temperature due to the mismatch in coefficients of thermal expansion (CTE) between Cu and BCB, with the polymer exhibiting a higher CTE. The overall dishing depth ($\Delta_{dishing}$) was determined as the sum of these two contributions, expressed as $\Delta_{dishing} = \Delta_{mechanical} + \Delta_{thermal}$. The dishing depth was obtained as 236.4 nm from the COMSOL modeling, and the material properties used in the calculation are summarized in Table S2. Since this value corresponds to the condition where the polymer interfaces of the top and bottom chips are just in contact, the target dishing depth during the polishing experiment was set slightly smaller than the calculated value, at 230 nm, to ensure interfacial contact while applying appropriate stress in the polymer region.

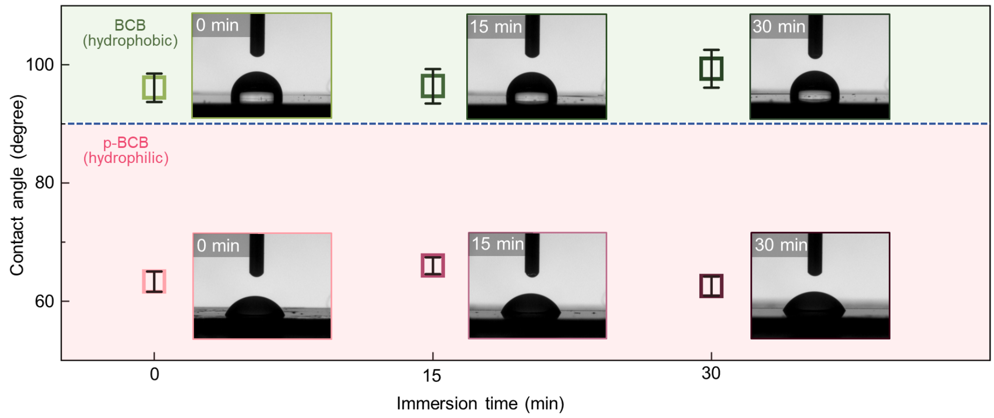

Figure S10. Contact angle analysis of BCB and p-BCB after slurry immersion. BCB consistently exhibited hydrophobicity, while p-BCB showed hydrophilicity, with no measurable change after immersion in slurry for 0, 15, and 30 minutes. These results confirm that surface energy and wettability of both polymers remain unaffected by the slurry exposure (n = 3, where n is the number of experiments conducted for generating statistical data).

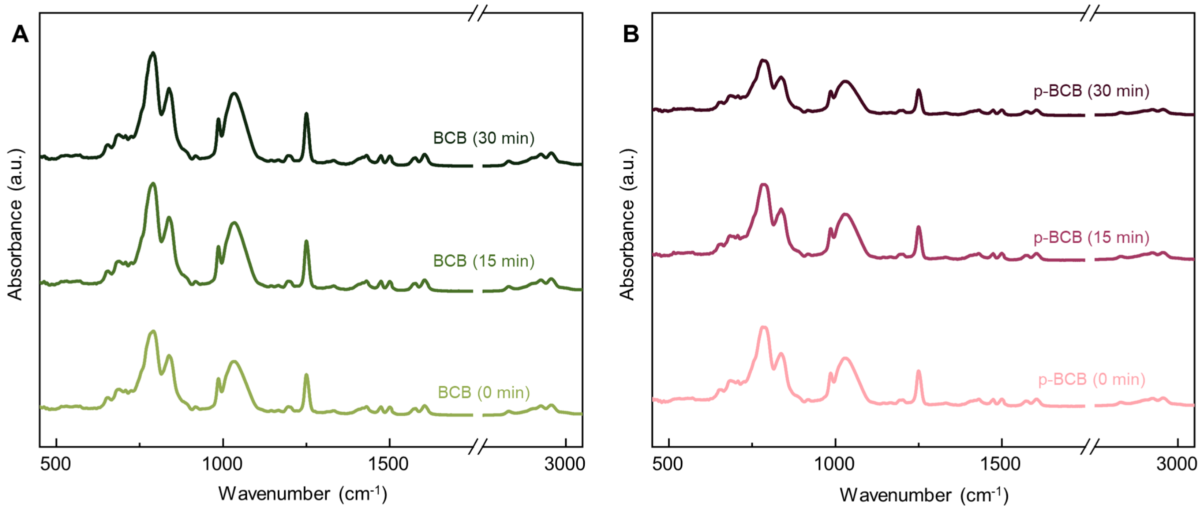

Figure S11. Fourier transform infrared spectroscopy (FTIR) spectra of (A) BCB and (B) p-BCB after slurry immersion. Characteristic molecular vibration peaks of both polymers were maintained without the appearance of new absorption bands or intensity variations, demonstrating that no bond cleavage or formation of new functional groups occurred after immersion in slurry for up to 30 minutes.

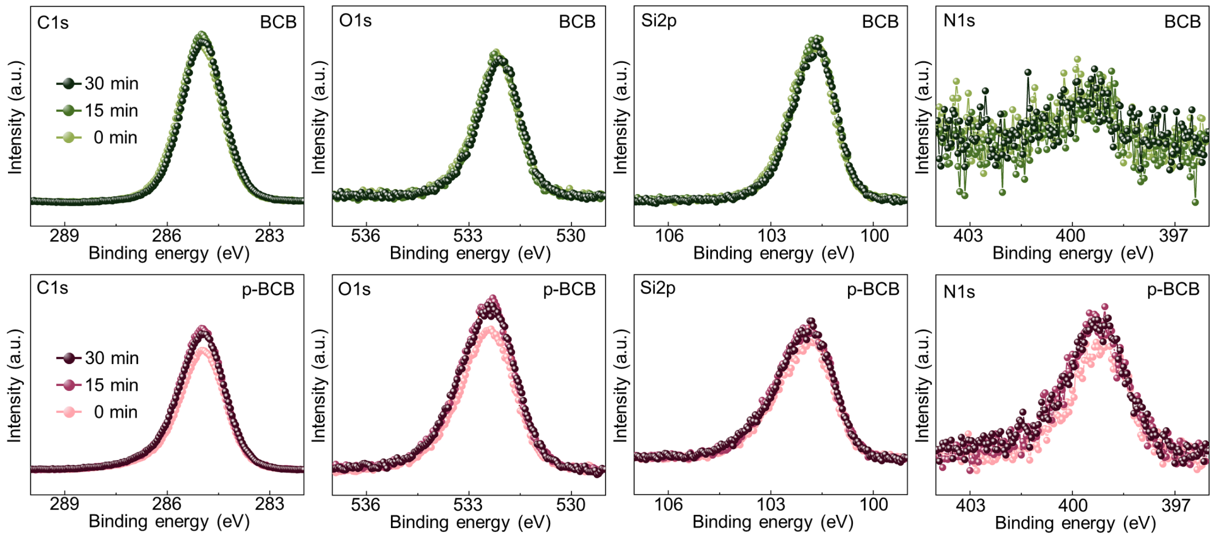

Figure S12. X-ray photoelectron spectroscopy (XPS) spectra of BCB and p-BCB after slurry immersion. XPS spectra of C1s, O1s, Si2p, and N1s remained unchanged with immersion time (0, 15, 30 minutes), confirming that the surface chemical composition and bonding states of the polymers were preserved and not modified by slurry exposure.

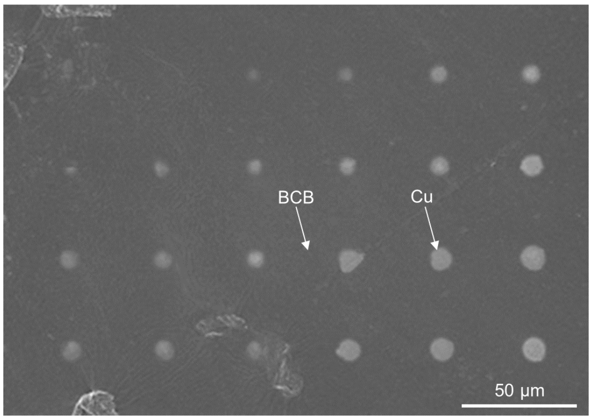

Figure S13. SEM image of the Cu/BCB surface after 900 s of polishing without plasma treatment. The Cu patterns became exposed only at a later stage of polishing, indicating the low material removal rate (MRR) of the pristine BCB layer (3.1 nm/s). Abrasive particle trapping occurred on the untreated BCB surface, during which micro-scratches formed and material removal proceeded non-uniformly across the wafer.

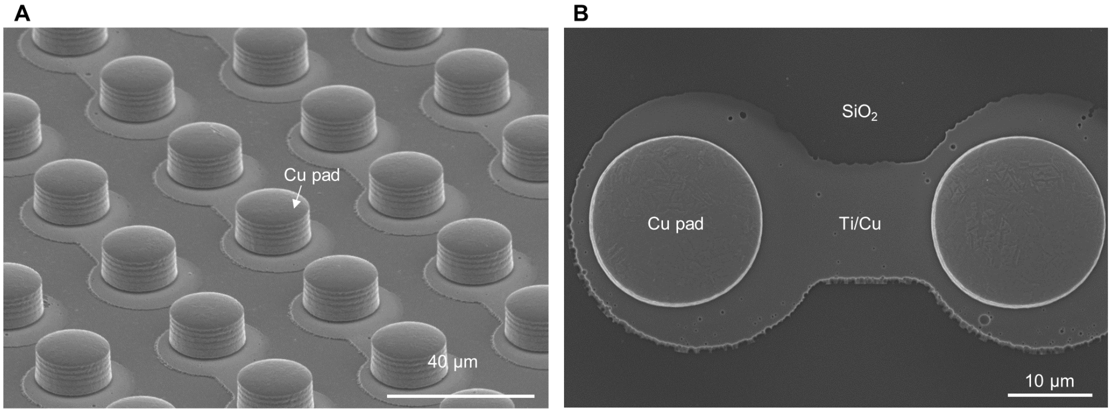

Figure S14. SEM images of the Cu pad-patterned wafer before BCB coating. (A) Tilted view showing an array of cylindrical Cu pads. (B) Top view of the Cu pad structure, where a Ti/Cu (1 μm) layer is deposited beneath the Cu pads to form a daisy chain structure.

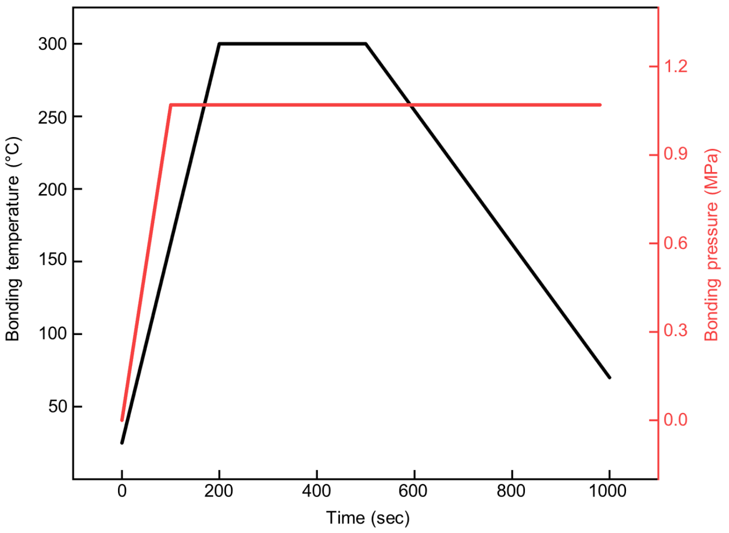

Figure S15. Bonding temperature and pressure profiles during the hybrid bonding process. Although bonding in this study was performed at 300 °C, the bonding behavior of BCB is known to depend on both temperature and time. Thus, bonding can potentially be achieved at lower temperatures by extending the duration, offering greater flexibility for integration schemes with tighter thermal budgets.

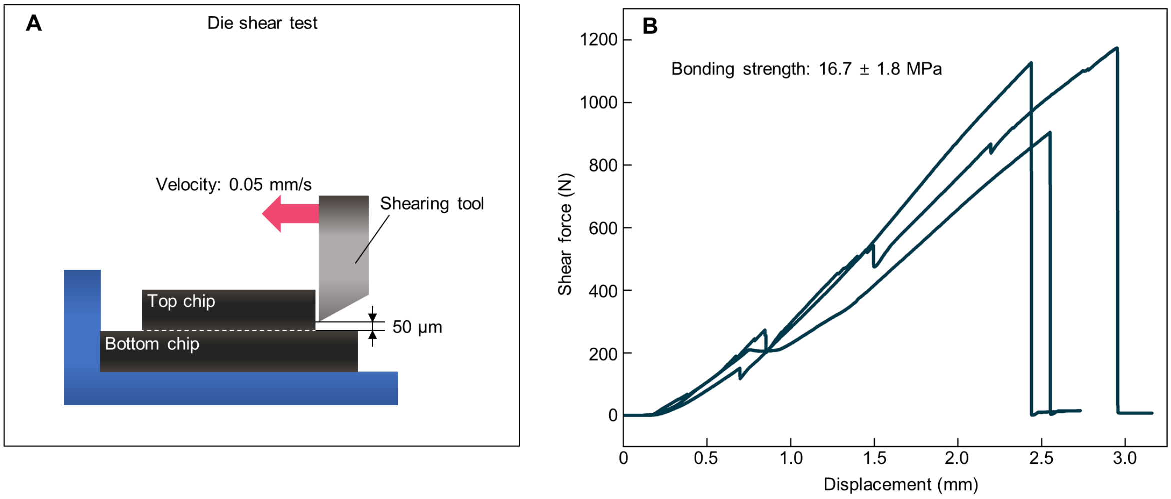

Figure S16. Die shear test for evaluating the bonding strength of the fabricated sample. (A) Schematic illustration of the die shear test setup using a UMT TriboLab system (Bruker). A shearing tool was applied to the top chip at a constant velocity of 0.05 mm/s, with a clearance of approximately 50 μm from the bottom chip. (B) Shear force-displacement curves obtained from the test. The average bonding strength, calculated by dividing the maximum shear force by the bonded area, was measured to be 16.7 ± 1.8 MPa, confirming the mechanical reliability of the bonded structure (n = 3, where n is the number of experiments conducted for generating statistical data).

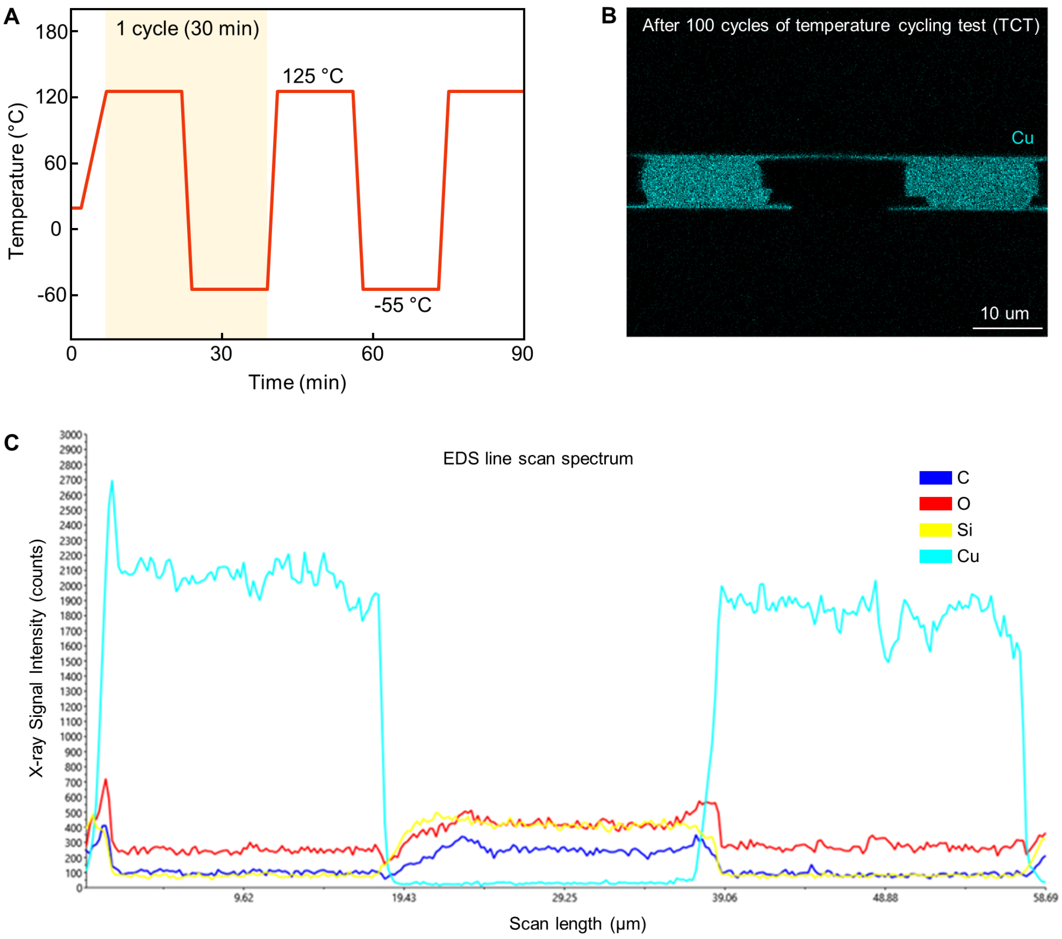

Figure S17. Evaluation of long-term thermal reliability of the Cu/BCB hybrid bonding interface through temperature cycling test (TCT). (A) Temperature profile of the TCT, where the sample was held at 125 °C and -55 °C for 15 min each, constituting one 30 min cycle. A total of 100 cycles was performed using the OST-TST-E010 system (Osung ST). (B) EDS mapping of the Cu/BCB interface after 100 TCT cycles. (C) EDS line scan spectrum of the corresponding region. No Cu diffusion into the BCB region was observed after 100 cycles. All experiments were conducted under a relative humidity of 40-60%.

Table S1. Comparative properties of polymer dielectrics (BCB, PI, PBO). Polyimide (PI) provides excellent insulation and thermal stability, but its high moisture absorption compromises long-term reliability. Polybenzoxazole (PBO) shows favorable dielectric characteristics but suffers from a high coefficient of thermal expansion (CTE), low mechanical rigidity, and relatively high moisture absorption, all of which limit its reliability. In contrast, BCB combines a low dielectric constant (Dk) and dissipation factor (Df) with low moisture absorption and a relatively low CTE, offering superior electrical performance and better thermal expansion matching with metal interconnects. These advantages make BCB the most suitable insulating polymer for ensuring both high-frequency performance and long-term reliability. In this study, BCB (Cyclotene 3000, Dow) was employed. Since material property data were available for the Cyclotene 4000 series, those values were referenced here. According to the technical datasheet, the 3000 series is designed for dry etching, while the 4000 series is photo-patternable, however, both series share the same polymer backbone and thus exhibit identical bulk material properties. (Data referenced from Nimbalkar et al., 2023; main text reference [47]).

Parameter	BCB	PI	PBO
Dielectric constant (Dk)	2.7	3.3	3.1
Dissipation factor (Df)	0.008	0.002	0.010
Elastic modulus [GPa]	2.9	2.45	1.8
Moisture absorption [wt%]	< 0.2	2 - 3	1.5
Coefficient of thermal expansion (CTE) [ppm/K]	42	40	80
Curing temperature (°C)	250	300	250
Product name (Manufacturer)	Cyclotene 4000 (Dow)	PI-2574 (HD Microsystems)	HD-8930 PBO (HD Microsystems)

Table S2. Material properties of Cu and BCB used in the COMSOL simulation.

**Parameter**	**Cu**	**BCB**
Young's modulus (E) [GPa]	120	2.9
Poisson's ratio (ν)	0.34	0.34
Density (ρ) [kg/m^3]	8960	1050
Coefficient of thermal expansion (α) [$10^{-6}\cdot$1/K]	16.5	52
Heat capacity (C_p) [J/(kg·K)]	384	2180
Thermal conductivity (κ) [W/(m·K)]	401	0.29

Table S3. Summary of constants used in the CMP modeling of Cu/BCB structures. k_{pad} is the stiffness of the polishing pad, and α is the pressure redistribution factor that accounts for surface height differences between Cu and BCB regions. $K_{p,\,Cu}$ and $K_{p,BCB}$ represent the Preston coefficients for Cu and BCB, respectively. Two different values are provided for $K_{p,BCB}$ to distinguish between the pristine and p-BCB surfaces. In the dishing model, the coefficient for p-BCB was applied from 180 s to 420 s, after which the coefficient for pristine BCB was used from 420 s to 900 s, reflecting the transition in surface condition as the p-BCB layer was removed during polishing.

Constants	Values
Stiffness of the polishing pad (k_{pad})	8.027×10^{10} N/m^3
Pressure redistribution factor (α)	3.1×10^5 N/m
Preston coefficients for Cu ($K_{p,\,Cu}$)	6.35×10^{-4} Pa^{-1}
Preston coefficient for untreated BCB ($K_{p,BCB}$)	1.02×10^{-3} Pa^{-1}
Preston coefficient for p-BCB ($K_{p,p-BCB}$)	2.92×10^{-3} Pa^{-1}

Supplementary Text

Theoretical Model for Dishing Behavior in Cu/BCB CMP

The nominal pressures applied to the Cu (p_{Cu}) and BCB (p_{BCB}) regions can be expressed as,

$$p_{Cu} = k_{pad}\{T_{pad} - (h - h_{Cu}\ ) + \frac{\Delta F}{l_{Cu}}\} \tag{1}$$

$$p_{BCB} = k_{pad}\{T_{pad} - (h - h_{BCB}\ ) - \frac{\Delta F}{l_{BCB}}\} \tag{2}$$

Here, k_{pad} denotes the stiffness of the polishing pad. The term ΔF represents the redistributed pressure arising from the height differences between the Cu and BCB regions. The redistributed pressure term is proportional to the difference in local surface heights, scaled by a redistribution factor α, and is expressed as,

$$\Delta F = \alpha(h_{Cu} - h_{BCB}) \tag{3}$$

The specific values of the constants used in the model are summarized in Table S3. The total applied pressure P applied to the entire system satisfies the following condition,

$$p_{Cu} \cdot l_{Cu} + p_{BCB} \cdot l_{BCB} = P \tag{4}$$

The MRR in each region follows Preston's equation and is expressed as,

$$\frac{dh_{Cu}}{dt} = K_{p,\,Cu} \cdot p_{Cu} \cdot v \tag{5}$$

$$\frac{dh_{BCB}}{dt} = K_{p,BCB} \cdot p_{BCB} \cdot v \tag{6}$$

Here, v is the relative velocity between the polishing pad and the wafer surface, and $K_{p,\,Cu}$, $K_{p,BCB}$ are the Preston coefficients for Cu and BCB, respectively. Notably, the value of $K_{p,BCB}$ varies depending on whether the BCB surface is p-BCB or pristine, reflecting differences in mechanical properties introduced by the surface modification.

Finally, the time-dependent dishing $D(t)$ is defined as the difference in surface height between the Cu and BCB regions, and expressed as follows.

$$D(t) = h_{Cu}(t) - h_{BCB}(t) \tag{7}$$
